# Identifying potential biomarkers in hepatitis B virus infection and its response to the antiviral therapy by integrated bioinformatic analysis

**DOI:** 10.1111/jcmm.16655

**Published:** 2021-05-26

**Authors:** Yi He, Yingzhi Zhou, Huimin Wang, Jingyang Yin, Yunan Chang, Peng Hu, Hong Ren, Hongmei Xu

**Affiliations:** ^1^ Department of infection National Clinical Research Center for Child Health and Disorders Ministry of Education Key Laboratory of Child Development and Disorders Children’s Hospital of Chongqing Medical University Chongqing China; ^2^ Chongqing Key Laboratory of Child Infection and Immunity Chongqing Medical University Chongqing China; ^3^ Chongqing People’s Hospital Chongqing Medical University Chongqing China; ^4^ Department of Infectious Diseases Key Laboratory of Molecular Biology for Infectious Diseases (Ministry of Education) Institute for Viral Hepatitis The Second Affiliated Hospital Chongqing Medical University Chongqing China

**Keywords:** bioinformatic analysis, chronic hepatitis B, immune cell infiltration, lncRNA, miRNA, therapeutics

## Abstract

The antiviral treatment efficacy varies among chronic hepatitis B (CHB) patients and the underlying mechanism is unclear. An integrated bioinformatics analysis was performed to investigate the host factors that affect the therapeutic responsiveness in CHB patients. Four GEO data sets (GSE54747, GSE27555, GSE66698 and GSE66699) were downloaded from the Gene Expression Omnibus (GEO) database and analysed to identify differentially expressed genes(DEGs). Enrichment analyses of the DEGs were conducted using the DAVID database. Immune cell infiltration characteristics were analysed by CIBERSORT. Upstream miRNAs and lncRNAs of hub DEGs were identified by miRWalk 3.0 and miRNet in combination with the MNDR platform. As a result, seventy‐seven overlapping DEGs and 15 hub genes were identified including CCL5, CXCL9, MYH2, CXCR4, CD74, CCL4, HLA‐DRB1, ACTA1, CD69, CXCL10, HLA‐DRB5, HLA‐DQB1, CXCL13, STAT1 and CKM. The enrichment analyses revealed that the DEGs were mainly enriched in immune response and chemokine signalling pathways. Investigation of immune cell infiltration in liver samples suggested significantly different infiltration between responders and non‐responders, mainly characterized by higher proportions of CD8+ T cells and activated NK cells in non‐responders. The prediction of upstream miRNAs and lncRNAs led to the identification of a potential mRNA‐miRNA‐lncRNA regulatory network composed of 2 lncRNAs (H19 and GAS5) and 5 miRNAs (hsa‐mir‐106b‐5p, hsa‐mir‐17‐5p, hsa‐mir‐20a‐5p, hsa‐mir‐6720‐5p and hsa‐mir‐93‐5p) targeting CCL5 mRNA. In conclusion, our study suggested that host genetic factors could affect therapeutic responsiveness in CHB patients. The antiviral process might be associated with the chemokine‐mediated immune response and immune cell infiltration in the liver microenvironment.

## 1 INTRODUCTION

More than 250 million people worldwide are chronically infected with hepatitis B virus (HBV), which can progress to chronic hepatitis B (CHB), liver cirrhosis, hepatocellular carcinoma and other HBV‐related complications.^1^ Despite the availability of antiviral drugs, many CHB patients are not thoroughly cured. The efficacy of antiviral therapy varies greatly from a complete response (virus clearance) to lack of response (persistent high viral load). This response variation probably results from the complicated interaction of the host immune response with the virus, in which both host factors and viral factors play important roles.^2^ Recent studies have led to increased attention to the roles of host factors in the antiviral treatment response than before. In HBV‐infected cell models, the cell‐intrinsic interferon (IFN) pathway activation of the host was found to be more important than the viral genotype in determining the antiviral efficacy of IFN‐α.^3^ Compared to responders of antiviral treatment, the mRNA and protein levels of IFITM2‐a blocker of IFN pathway activation were found to be significantly higher in both the liver and peripheral blood samples of non‐responders.^4^ In contrast, the expression level of ISG20 (an IFN‐stimulated gene) in liver biopsy samples was found to be significantly lower in non‐responders than in responders.^5^ Previous studies suggested that host genetic factors and immune status were probably associated with the therapeutic responsiveness of CHB, but the underlying mechanism is not well understood.

Bioinformatics analysis of gene expression profiles is a promising method to comprehensively identify different genetic factors and pathways of a certain pathophysiological process. Previous studies have explored the differentially expressed genes (DEGs) of HBV‐related liver diseases, including liver fibrosis, liver failure and hepatocellular carcinoma, using bioinformatics analysis methods,^6^, ^7^, ^8^, ^9^ but few studies have explored the factors of anti‐HBV treatment response.^10^ Moreover, false‐positive rates in a single microarray analysis might lead to inaccuracy. To explore the host factors and the underlying mechanism leading to suboptimal therapeutic responsiveness, we downloaded 4 gene expression profiles (GSE54747, GSE27555, GSE66698 and GSE66699) from the Gene Expression Omnibus (GEO) database and performed an integrated bioinformatic analysis.

## 2 MATERIALS AND METHODS

### 2.1 Microarray data

The mRNA microarray data sets were downloaded from GEO database (http://www.ncbi.nlm.nih.gov/geo/). Our initial aim was to explore the key genes involved in therapeutic responsiveness of CHB patients. We searched for the potential GEO data sets according to the following inclusion criteria: (a) human liver specimens with histological diagnosis; (b) gene expression profiling of mRNA; (c) data sets concerning gene expression of liver tissues diagnosed as HBV‐positive and treated with antiviral therapies; (d) data sets enrolling CHB patients with hepatic decompensation, hepatocellular carcinoma, liver failure, solid organ or bone marrow transplantation, chronic immunosuppression or coinfection of other hepatotropic virus were excluded. After screening the titles and evaluating the full information of the potential data sets, 4 GEO data sets (GSE54747, GSE27555, GSE66698 and GSE66699) were found.

### 2.2 Identification of DEGs

The interactive web tool GEO2R (https://www.ncbi.nlm.nih.gov/geo/geo2r), provided by the GEO database, was utilized to screen DEGs. |log_2_FC| > 1 was set as the cut‐off standards and *P* < .05 was considered to indicate statistical significance. The adjustment of *P*‐values and the Benjamini and Hochberg method were applied to optimize the statistical power and control false discovery rate. Using a Venn diagram to overlap the DEGs of the 4 data sets, DEGs that commonly existed in more than 2 independent data sets were selected.

Gene Ontology (GO), Kyoto Encyclopedia of Genes and Genomes (KEGG) pathway enrichment analyses of DEGs.

GO database (http://www.geneontology.org) contains structured ontology or vocabularies that annotate genes and the biological function of these genes.^11^ KEGG database (http://www.genome.jp/kegg/) synthesizes information for functions and biological systems from large‐scale molecular data sets generated by high‐throughput experimental technologies.^12^ To analyse the function of DEGs, Database for Annotation, Visualization and Integrated Discovery(DAVID; https://david.ncifcrf.gov/) was utilized to conduct GO enrichment analyses (including biological process (BP), cellular component (CC) and molecular function (MF)) and identify the most enriched KEEG pathways. *P* < .01 was considered as statistically significant for screening. The enrichment analyses were performed for up‐regulated and down‐regulated genes separately.

### 2.3 Protein‐protein interaction (PPI) Network Construction

PPI networks were constructed by Search Tool for the Retrieval of Interacting Genes (STRING) online database (http://string‐db.org/). Interaction with a combined confidence score ≥0.4 was considered statistically significant. The result of STRING analysis was imported into Cytoscape v.3.7.1 to visualize the molecular interaction networks. The plugin Molecular Complex Detection (MCODE) (version 1.4.2) of Cytoscape was utilized to cluster the PPI network and find the densely connected region or hub modules based on topology. The criteria of cluster analysis were MCODE scores >5, degree cut‐off = 2, node score cut‐off = 0.2, Max depth = 100 and k‐score = 2. The functional analyses of genes involved in the hub modules were performed using DAVID.

### 2.4 Identification of hub genes

The hub genes were selected using the cytoHubba plugin of Cytoscape. By calculating the scores of 5 ranked methods separately, including degree, maximum neighbourhood component, radiality centrality, stress centrality and closeness centrality,^13^ the top 25 genes of each method were screened out. Then, by overlapping the top 25 genes of each method, the hub genes were identified. Next, the function of hub genes was analysed using DAVID. Finally, the biological process analysis of hub genes was visualized using Biological Networks Gene Oncology tool (BiNGO) (version 3.0.3)—a plugin of Cytoscape.

### 2.5 Immune cell infiltration evaluation

CIBERSORT is an analytical tool designed to predict the relative levels of 22 immune cell types from gene expression.^14^ Normalized gene expression data were uploaded to the CIBERSORT web portal (https://cibersort.stanford.edu). ‘Signature gene file’ was set as ‘LM22(22 immune cell types)’, the number of permutations was set to 100, and the analyses were performed under relative mode. The CIBERSORT *P*‐value reflects the statistical significance of the deconvolution results across all cell subsets and immune cell profiles of samples with a CIBERSORT *P* < .5 was considered to have significant fitting accuracy and was included in further analyses. Student's *t* test or LSD‐*t* test was used to analyse the differences of immune cell fractions between responders and non‐responders. Correlation analysis of immune cell proportions in all samples was performed using the HIPLOT web tool, which is a free online platform for data analysis (https://hiplot.com.cn/basic). Pearson correlation matrix was then constructed.

### 2.6 Prediction of related non‐coding RNAs(ncRNAs)

The DEGs were input into miRWalk3.0 database (http://mirwalk.umm.uni‐heidelberg.de/) to predict their targeted miRNAs. The searching conditions were set as follows: *P *< .05, experimentally validated and 3′UTR as the target gene binding region. Then, the selected miRNAs were uploaded to miRNet (https://www.mirnet.ca/miRNet/home.xhtml) to predict their upstream lncRNAs. The searching conditions were set as ‘Organism‐H.sapies’ and ‘targets‐lncRNAs’. Next, to verify the accuracy of the above prediction, Mammal ncRNA‐disease repository (MNDR) database was used to do an intersection with the above results. The MNDR online platform (http://www.rna‐society.org/mndr/home.html) was designed for efficient browsing the associations between ncRNA (including lncRNA, miRNA, piRNA, snoRNA) and diseases in mammals.^15^ By browsing the mesh terms or the disease ontology ‘hepatitis B/hepatitis B, chronic’, the disease‐related ncRNAs were downloaded from MNDR v3.1. In this study, we restricted the ‘species’ to ‘Homo sapiens’ and only included ncRNA‐disease associations that were evaluated as ‘Strong Evidence’ by MNDR v3.1 platform. The final result obtained from the intersection was further processed with Cytoscape.

### 2.7 Chemokine secretion assays by quantibody® array kit

Five potential serum biomarkers for predicting CHB treatment response, including CCL4, CCL5, CXCL9, CXCL10 and CXCL13, were selected for further confirmation in CHB patients receiving antiviral therapy. Therapeutic response was defined as HBeAg negativity, serum HBV DNA undetectable (<400 IU/mL), and normalization of serum ALT at the end of follow‐up in this study. CHB patients who were regularly followed up in Chongqing Children's Hospital were enrolled, excluding those with hepatic decompensation, hepatocellular carcinoma, liver failure, chronic immunosuppression or coinfection of other hepatotropic virus. A total of 27 CHB patients, consisting of 15 responders and 12 non‐responders to antiviral treatment, were included. Twelve patients completed 48 weeks of PegIFNα‐2a treatment and another 48 weeks of follow‐up (96 weeks of follow‐up in total). Fifteen patients received entecavir (ETV) monotherapy for 96 weeks.

Blood samples were obtained from the 27 patients at baseline and from 21 of them at 16 weeks of treatment. Serum samples were prepared by centrifugation at 1006.2 *g* for 20 minutes and stored at −80°C. A human chemokine quantibody® array kit (Raybiotech)—a multiplexed sandwich ELISA‐based quantitative array platform, was used per the manufacturer's instructions. InnoScan 300 Microarray Scanner (Innopsys, arc d'Activités Activestre, 31 390 Carbonne‐France) was used for fluorescence detection. The Quantibody® array quantitatively measured the expression of a series of chemokines including CCL4, CCL5, CXCL9, CXCL10, and CXCL13. The levels of chemokines expression were compared between responders and non‐responders at baseline and at 16 weeks of treatment. Data were analysed with SPSS 26.0 (SPSS, Inc Chicago, IL, United States). Comparisons between the 2 groups were performed using Student's *t* test or Mann‐Whitney test for continuous variables and Fisher's exact test for categorical variables. Written informed consent was obtained from all patients prior to beginning the study. This study was approved by the ethics committee of Chongqing Medical University.

## 3 RESULTS

### 3.1 Identification of DEGs

**FIGURE 1:**
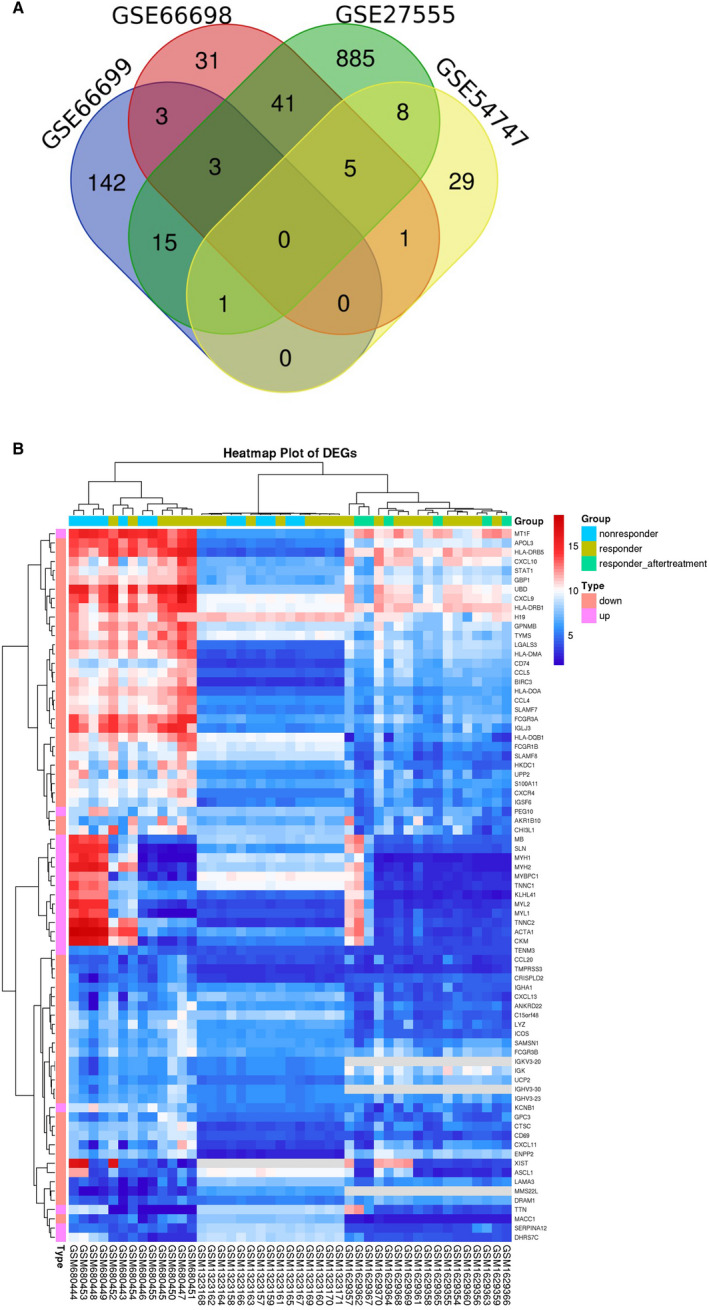
A, Venn diagram: differential expressed genes (DEGs) were selected with a fold change >1 and *P*‐value <0.05 among the mRNA expression profiling sets GSE66698, GSE66699, GSE54747 and GSE27555. The 4 data sets showed an overlap of 77 DEGs. B, Heat map of the selected 77 DEGs:blue indicates a relatively low expression, and red indicates a relatively high expression

According to the search criteria for gene expression microarrays, 4 GEO data sets (GSE54747, GSE27555, GSE66698 and GSE66699) were included in our study. The GSE54747 data set contained 9 liver biopsy samples of responders and 6 samples of non‐responders obtained before peginterferon (PEG‐IFN) combined with adefovir therapy.^16^ The GSE27555 data set contained 7 samples of responders and 6 samples of non‐responders obtained before IFN‐α therapy.^17^ The GSE66698 data set contained 10 pre‐treatment samples and 7 post‐treatment samples from 10 IFN‐α therapy responders.^18^ The GSE66699 data set contained 2 pooled samples of 11 responders and 11 non‐responders before IFN‐α therapy.^18^ After differential expression analysis conducted by GEO2R, a total of 1,164 DEGs (44 in GSE54747, 85 in GSE66698, 164 in GSE66699 and 958 in GSE27555, respectively) were identified. After overlapping the DEGs among the 4 data sets, 77 DEGs shared by ≥2 data sets were identified, as shown in the Venn diagram (Figure 1A). The 77 overlapping DEGs consisting of 58 down‐regulated DEGs and 19 up‐regulated DEGs were selected for the subsequent analyses. After standardization, gene expression microarray data of the selected DEGs in each sample are shown in the heat map (Figure 1B). Table S1 shows the details of the selected 77 DEGs of the 4 data sets.

### 3.2 Enrichment analyses of DEGs

**FIGURE 2:**
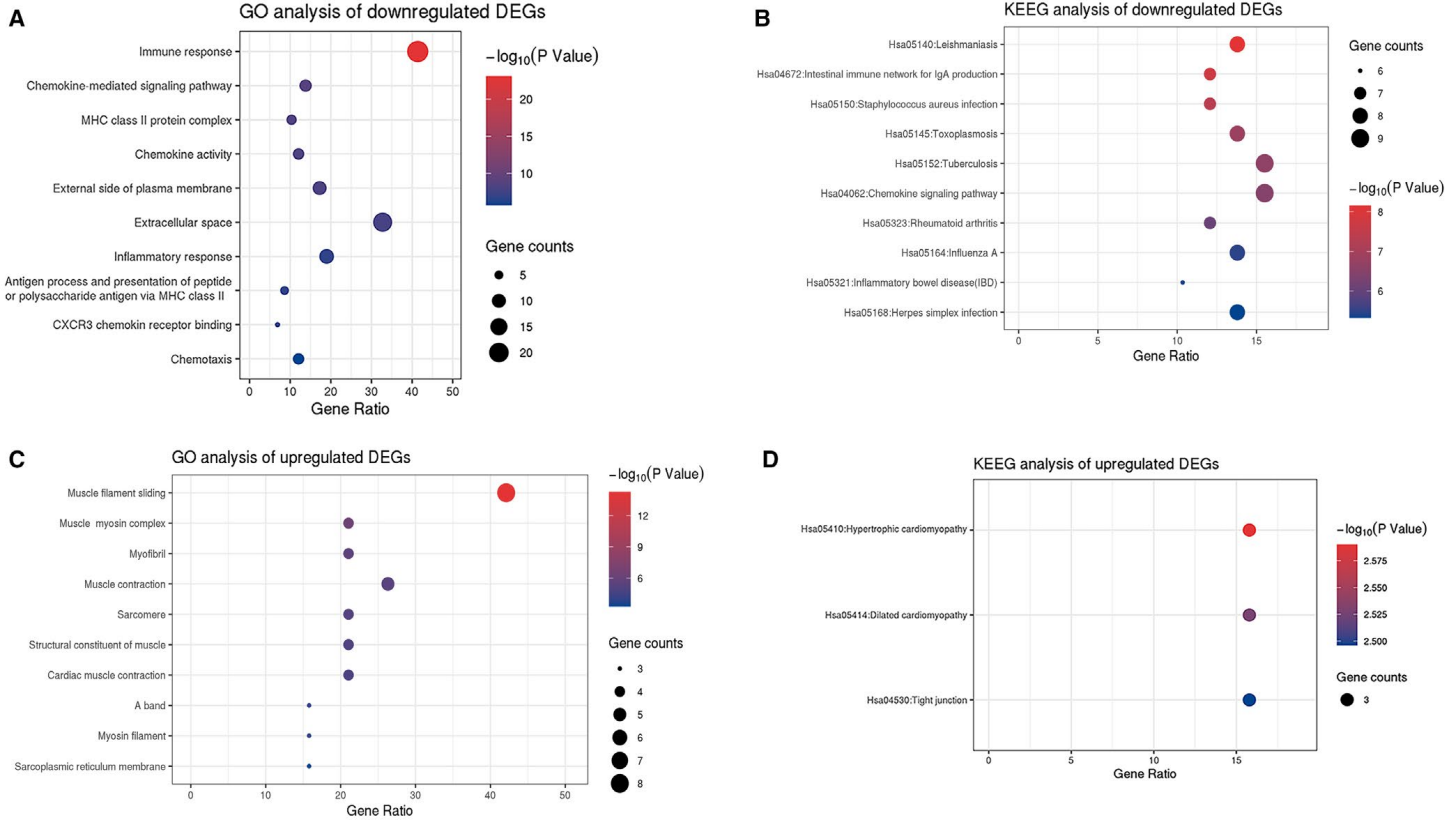
Gene Ontology (GO) and Kyoto Encyclopedia of Genes and Genomes (KEGG) pathway enrichment analysis of up‐regulated and down‐regulated differential expressed genes(DEGs). A, Top 10 enriched GO terms of downregulated DEGs ranked by *P*‐value. B, Top 10 enriched KEEG pathways of down‐regulated DEGs ranked by *P*‐value. C, Top 10 enriched GO terms of up‐regulated DEGs ranked by *P*‐value. D, Enriched KEEG pathways of up‐regulated DEGs ranked by *P*‐value

To predict the biological functional and pathways of DEGs, enrichment analyses were performed using DAVID. The significantly enriched gene sets were set at a default cut‐off as *P* < .01. To determine the most significant GO terms and KEGG pathways, all enriched terms were sequenced by *P*‐value. The top 10 terms with the lowest *P*‐values are shown in Figure 2. Then, the down‐regulated and up‐regulated DEGs were analysed separately. The down‐regulated DEGs were most significantly enriched in immune response (GO:0006955); KEGG pathway analysis showed that the down‐regulated DEGs were most significantly enriched in Chemokine signalling pathway (hsa04062) apart from some auto‐immune diseases and infectious diseases (Figure 2A and B). The up‐regulated DEGs were most significantly enriched in muscle filament sliding (GO: 0030049) and hypertrophic cardiomyopathy (hsa05410; Figure 2C and D). Next, the enrichment analyses of GO and KEGG pathway were sorted by the counts of involved genes, as shown in Table S2. For the 58 down‐regulated DEGs, changes in BP were mainly enriched in immune response(GO:0006955) and inflammatory response(GO:0006954); changes in CC were mainly enriched in extracellular space (GO:0005615), and extracellular exosome (GO:0070062); changes in MF were mainly enriched in chemokine activity (GO:0008009) and heparin binding (GO:0008201); changes in KEGG pathways were mainly enriched in Chemokine signalling pathway (hsa04062) and Cytokine‐cytokine receptor interaction (hsa04060). For the 19 up‐regulated DEGs, changes in BP were mainly enriched in muscle filament sliding (GO:0030049); changes in CC were mainly enriched in cytosol (GO:0005829); changes in MF were mainly enriched in calcium ion binding (GO:0005509); changes in KEGG pathways were mainly enriched in hypertrophic cardiomyopathy (hsa05410).

### 3.3 PPI network construction and module analyses

**FIGURE 3:**
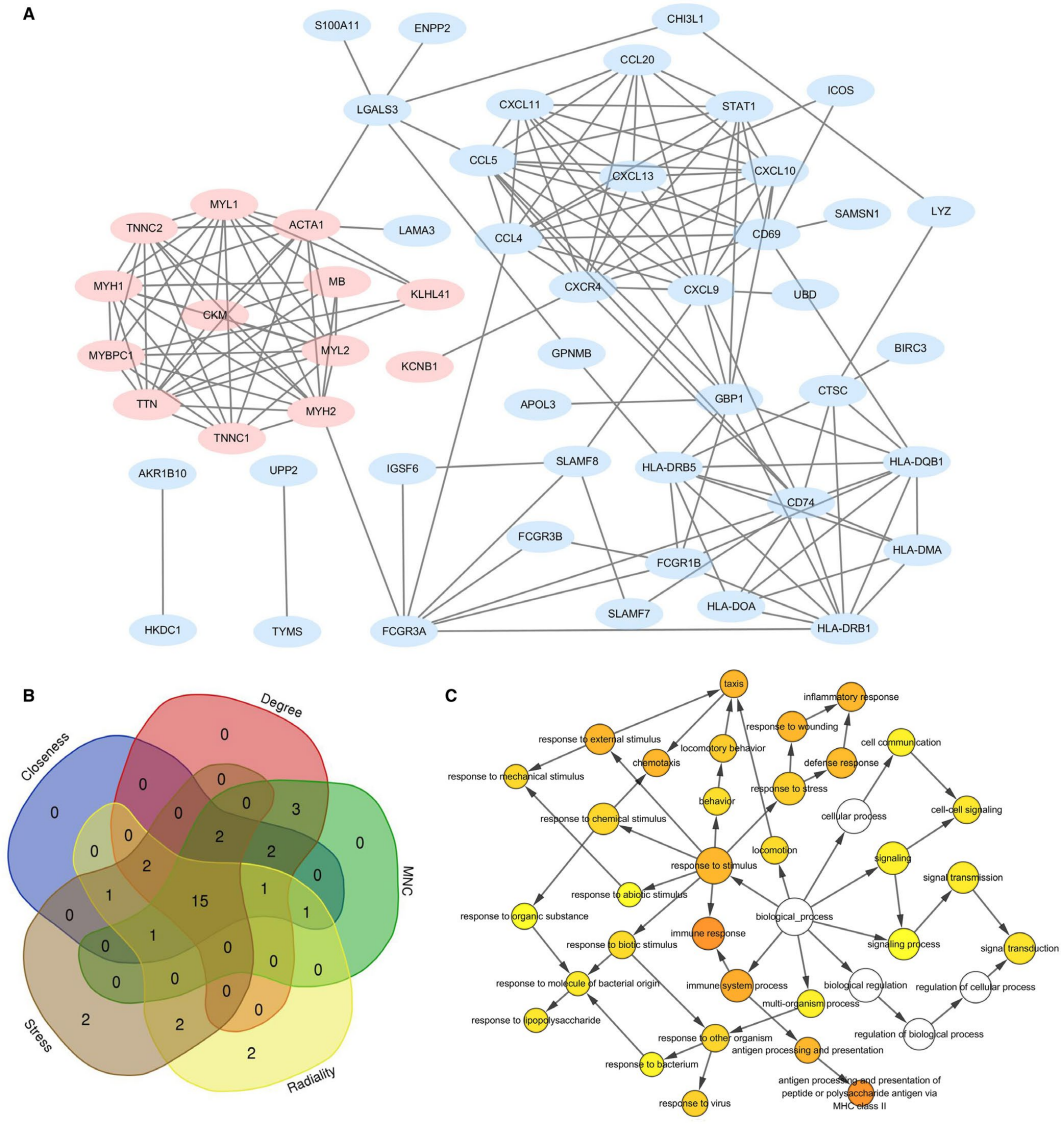
A, The protein‐protein interaction(PPI) network of the differentially expressed genes (DEGs) was constructed using Cytoscape. B, Overlapping the first 25 genes in the five classification methods of cytoHubba to identify hub genes. C, The biological process(BP) analysis of hub genes was constructed using BiNGO. The colour depth of nodes refers to the corrected *P*‐value of ontologies. The size of nodes refers to the numbers of genes that are involved in the ontologies. *P* <.01 was considered statistically significant

Based on the data from STRING database analysis, the PPI network of the DEGs was visualized using Cytoscape (Figure 3A). The hub modules in the network were identified using MCODE, and the genes involved in the hub modules were analysed using DAVID. Table 1 shows that genes in these hub modules were mainly enriched in the following BPs: muscle filament sliding, chemokine‐mediated signalling pathway and immune response.

**TABLE 1:** Hub modules and top biological process of genes involved in each module

Module	Score	Node	Edge	Node ID	Seed	Top biological process	Gene count	*P*‐value
1	9.600	11	48	CKM,MYL1,ACTA1,MB, MYL2,MYH2,TNNC1,TTN, MYBPC1,MYH1,TNNC2	CKM	Muscle Filament Sliding	8	2.0E‐17
2	9.333	10	42	CXCL13,CCL20,CXCL10, STAT1,CD69,CXCL9,CXCR4, CCL4,CCL5,CXCL11	CXCL13	Chemokine‐mediated signalling pathway	8	6.4E‐16
3	6.500	9	26	CD74,GBP1,CTSC,HLA‐DQB1,HLA‐DMA,HLA‐DRB1,HLA‐DOA,HLA‐DRB5,FCGR1B	CD74	Immune response	8	4.6E‐11

### 3.4 Hub genes identification and analyses

The top 25 genes were selected by the 5 classification methods in cytoHubba (Table S3). Finally, 15 hub genes were obtained by overlapping the top 25 genes of each method, as shown in Figure 3B. The 15 hub genes were sequenced according to the overall rankings of 5 classification methods: CCL5 (C‐C motif chemokine ligand 5), CXCL9 (C‐X‐C motif chemokine ligand 9), MYH2 (myosin heavy chain 2), CXCR4 (C‐X‐C motif chemokine receptor 4), CD74 (CD74 molecule), CCL4 (C‐C motif chemokine ligand 4), HLA‐DRB1 (major histocompatibility complex, class II, DR beta 1), ACTA1 (actin, alpha 1, skeletal muscle), CD69 (CD69 molecule), CXCL10 (C‐X‐C motif chemokine ligand 10), HLA‐DRB5 (major histocompatibility complex, class II, DR beta 5), HLA‐DQB1 (major histocompatibility complex, class II, DQ beta 1), CXCL13 (C‐X‐C motif chemokine ligand 13), STAT1 (signal transducer and activator of transcription 1) and CKM (creatine kinase, M‐type). The primary enriched GO terms and KEEG pathways analysed by DAVID were summarized in Table 2. Hub genes involved in the top 3 GO terms and KEEG pathways were all down‐regulated genes. The BP analysis of the hub genes using BiNGO is shown in Figure 3C. The most significant BPs of hub genes were immune response, antigen processing and presentation of peptide or polysaccharide antigen via MHC class II, immune system process, response to stimulus, antigen processing and presentation, inflammatory response, response to wounding, response to external stimulus, taxis and chemotaxis.

**TABLE 2:** Hub genes and top GO terms and KEEG pathways of enrichment analyses

Category	Term	Count	*P*‐Value	Genes
BP	GO:0 006 955~immune response	9	3.85E‐10	CXCL10, CD74, HLA‐DRB5, CXCL9, CCL5, CCL4, CXCL13, HLA‐DRB1, HLA‐DQB1
	GO:0 070 098~chemokine‐mediated signalling pathway	6	2.28E‐9	CXCL10, CXCL9, CCL5, CCL4, CXCR4, CXCL13
	GO:0 002 381~immunoglobulin production involved in immunoglobulin mediated immune response	3	6.45E‐6	HLA‐DRB5, HLA‐DRB1, HLA‐DQB1
CC	GO:0 009 897~external side of plasma membrane	6	3.82E‐7	CXCL10, CD74, HLA‐DRB5, CXCL9, CD69, HLA‐DRB1
	GO:0 042 613~MHC class II protein complex 4	4	5.51E‐7	CD74, HLA‐DRB5, HLA‐DRB1, HLA‐DQB1
	GO:0 071 556~integral component of lumenal side of endoplasmic reticulum membrane	4	1.30E‐6	CD74, HLA‐DRB5, HLA‐DRB1, HLA‐DQB1
MF	GO:0 008 009~chemokine activity	5	6.14E‐8	CXCL10, CXCL9, CCL5, CCL4, CXCL13
	GO:0 048 248~CXCR3 chemokine receptor binding	3	6.38E‐6	CXCL10, CXCL9, CXCL13
	GO:0 031 730~CCR5 chemokine receptor binding	3	1.78E‐5	STAT1, CCL5, CCL4
KEGG	hsa04062:Chemokine signalling pathway	7	2.91E‐7	CXCL10, CXCL9, STAT1, CCL5, CCL4, CXCR4, CXCL13
	hsa04620:Toll‐like receptor signalling pathway	5	2.40E‐5	CXCL10, CXCL9, STAT1, CCL5, CCL4
	hsa04060:Cytokine‐cytokine receptor interaction	6	3.41E‐5	CXCL10, CXCL9, CCL5, CCL4, CXCR4, CXCL13

Abbreviations: BP, biological process; CC, cellular component; GO, Gene Ontology; KEGG, Kyoto Encyclopedia of Genes and Genomes; MF, molecular function.

### 3.5 Immune cell infiltration characterization

**FIGURE 4:**
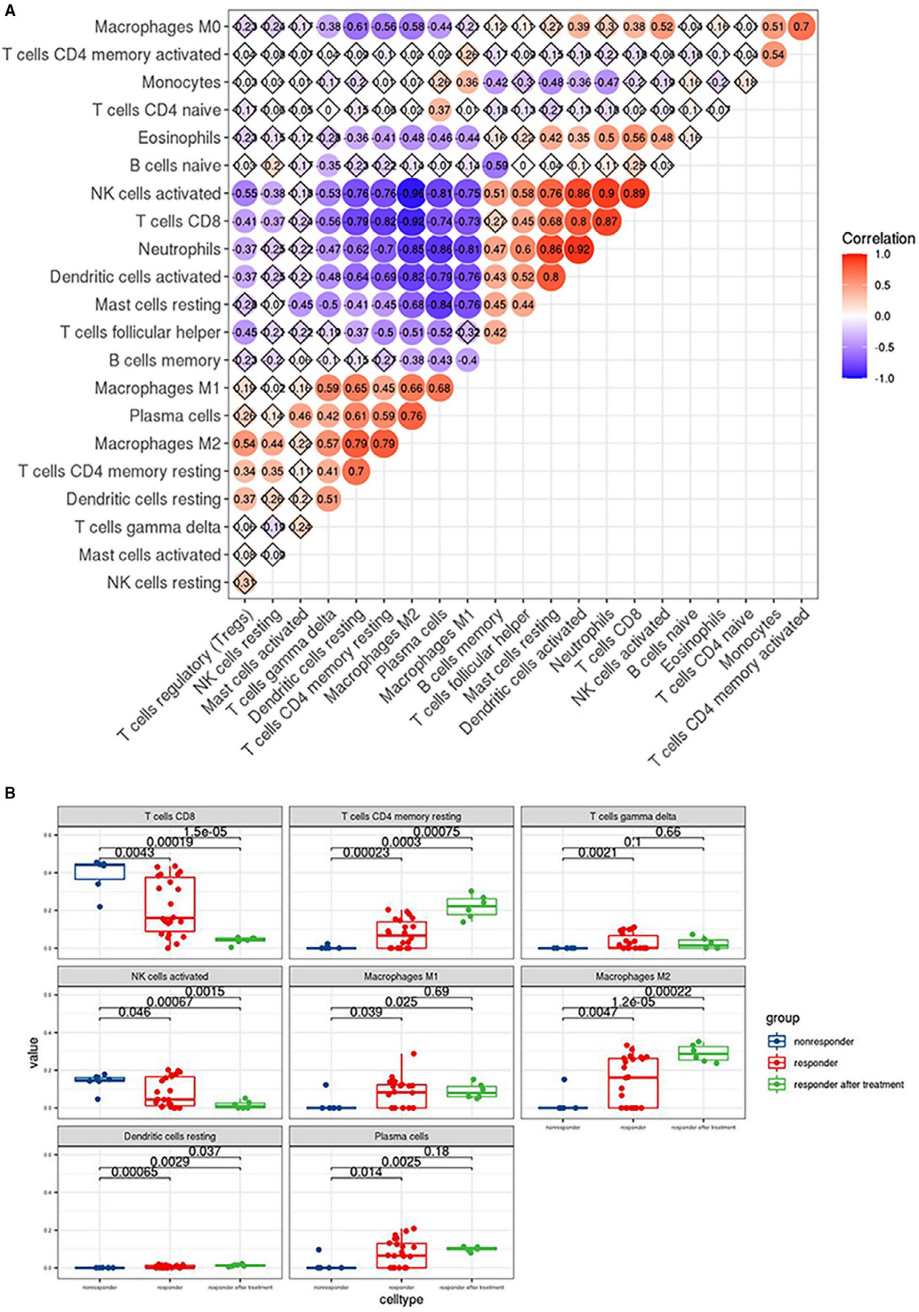
A, Correlation matrix of the immunocyte proportions in all samples. Red and blue colour represent positive and negative correlations, respectively; numbers in the nodes represent related coefficient(RC)(|RC|<0.3 no correlation; 0.3≤|RC|<0.5 low correlation; 0.5≤|RC|<0.8 moderate correlation;|RC|≥0.8 significant correlation). B:Differences in proportions of each immune cell type in responders and non‐responders. Blue, red and green colour represent liver samples of responders before treatment, responders after treatment and non‐responders before treatment. Numbers above any two boxs represent *P*‐values (*P*‐value <0.05 was considered to be significantly different)

Considering the above results, we speculated that the immune response was crucial in the DEGs pathway. Therefore, we decided to further explore the immune cell infiltration characterization of the liver samples. We investigated the liver samples that matched the requirements of the CIBERSORT algorithm, including 45 samples of 26 responders (including 26 samples before treatment and 6 samples after treatment) and 13 non‐responders. The immune cell composition for each sample predicted by CIBERSORT is summarized in Table S4. Among the 45 liver samples, 35 liver samples met the criteria with a *P *< .5 and were chosen for the subsequent analyses. Correlation analysis revealed the following significantly negative correlations: macrophages M2 vs NK cells activated (related coefficient (RC) = −0.96), macrophages M2 vs T cells CD8^+^ activated (RC = −0.92) and macrophages M2 cell vs neutrophils (RC = −0.85). In contrast, the following significantly positive correlations were found: neutrophils vs dendritic cells activated (RC = 0.92), neutrophils vs NK cells activated (RC = 0.90) and NK cells activated vs T cells CD8^+^ (RC = 0.89). Correlations between various immune cell proportions are shown in Figure 4A. Comparing the immune cell proportions of liver samples between responders and non‐responders, 8 out of 22 kinds of immune cells were significantly different. (Figure 4B *P *< .05 was considered to be significantly different). Higher proportions of macrophages M1, macrophages M2, plasma cells, CD4^+^ T cells memory resting, T cells gamma delta and dendritic cells resting were detected in responders, while higher proportions of NK cells activated and T cells CD8^+^ were detected in non‐responders (*P* < .05). T cells follicular helper and B cells memory tended to be higher in responders, while T cells regulatory (Treg), neutrophils, dendritic cells activated, macrophages M0, B cells naive and mast cells resting tended to be higher in non‐responders, but the differences were not significant (Figure S1).

### 3.6 Prediction of related non‐coding RNAs (ncRNAs)

**FIGURE 5:**
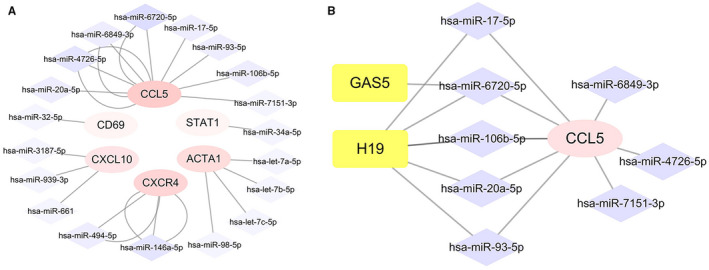
A, Interaction network between hub genes and its targeted miRNAs. Genes are coloured in pink; miRNAs are coloured in purple. Intensity of colour is adjusted according to the number of targeting lines. B, The mRNA‐miRNA‐lncRNA competing endogenous RNA (ceRNA) triple regulatory sub‐network associated with the effect and prognosis of antiviral therapy in hepatitis B. Genes are coloured in pink; miRNAs are coloured in purple; lncRNAs are coloured in yellow

Gene‐miRNA analyses were performed with miRWalk 3.0 software, and 5247 upstream miRNAs targeting the 15 hub genes were predicted. As previously mentioned, we only included miRNA‐gene interactions that have been validated by previously reported assays. After screening, 19 validated miRNAs targeting 6 hub genes (CCL5, ACTA1, CXCR4, STAT1, CXCL10 and CD69) were identified (Table 3). Figure 5A illustrates the interaction network of the validated miRNAs and genes. Next, we further predicted the lncRNAs that were potentially related to the selected miRNAs using miRNet database. A total of 946 lncRNAs were discovered. Finally, we searched for the ncRNAs related to hepatitis B on the MNDR platform, and a total of 47 miRNAs and 3 lncRNAs were found to be associated with hepatitis B with high reliability (Table S5). After cross‐linking ncRNAs predicted to be associated with the hub genes and those identified through the MNDR platform, 2 key lncRNAs(H19 and GAS5) targeting 5 key miRNAs (hsa‐mir‐106b‐5p, hsa‐mir‐17‐5p, hsa‐mir‐20a‐5p, hsa‐mir‐6720‐5p and hsa‐mir‐93‐5p) were discovered (Table 4). Based on the above gene‐miRNA analyses, CCL5 was found to be the most common targeted gene (Figure 5B).

**TABLE 3:** The validated miRNA‐mRNA pairs identified by miRWalk 3.0 database

miRNA ID	Gene symbol	Number of pairings	Binding region length	Longest consecutive pairings	Validated data source
hsa‐let‐7a‐5p	ACTA1	15	16	15	MIRT550902
hsa‐let‐7b‐5p	ACTA1	21	25	14	MIRT550901
hsa‐let‐7c‐5p	ACTA1	19	24	13	MIRT550900
hsa‐miR‐98‐5p	ACTA1	15	16	15	MIRT550892
hsa‐miR‐146a‐5p	CXCR4	19	27	9	MIRT000006
hsa‐miR‐146a‐5p	CXCR4	20	41	11	MIRT000006
hsa‐miR‐494‐5p	CXCR4	18	22	12	MIRT735294
hsa‐miR‐34a‐5p	STAT1	15	19	7	MIRT025278
hsa‐miR‐661	CXCL10	14	28	8	MIRT540505
hsa‐miR‐939‐3p	CXCL10	9	10	9	MIRT540515
hsa‐miR‐3187‐5p	CXCL10	15	21	7	MIRT540513
hsa‐miR‐32‐5p	CD69	17	21	8	MIRT728020
hsa‐miR‐20a‐5p	CCL5	16	23	9	MIRT685367
hsa‐miR‐4726‐5p	CCL5	20	26	10	MIRT518347
hsa‐miR‐4726‐5p	CCL5	18	23	13	MIRT518347
hsa‐miR‐6720‐5p	CCL5	22	36	15	MIRT685342
hsa‐miR‐6849‐3p	CCL5	18	25	9	MIRT685347
hsa‐miR‐17‐5p	CCL5	16	23	9	MIRT685368
hsa‐miR‐93‐5p	CCL5	17	23	10	MIRT685363
hsa‐miR‐106b‐5p	CCL5	15	21	10	MIRT685369
hsa‐miR‐7151‐3p	CCL5	15	19	8	MIRT518343

**TABLE 4:** The correlation between miRNA‐lncRNA pairs identified by cross‐linking miRNet and MNDR database

lncRNA ID	Disease Name	Scores by MNDR	Correlated miRNA	Experiment
H19	Hepatitis B, Chronic	0.999141	hsa‐mir‐106b‐5p	CLIP‐Seq
			hsa‐mir‐17‐5p	
			hsa‐mir‐20a‐5p	
			hsa‐mir‐6720‐5p	
			hsa‐mir‐93‐5p	
GAS5	Hepatitis B	0.982118	hsa‐mir‐6720‐5p	CLIP‐Seq

Taking all the 3 levels into consideration, we proposed a mRNA‐miRNA‐lncRNA triple subnetwork (Figure 5B). Classically, there are negative correlations between miRNAs and lncRNAs or mRNAs and inversely positive associations between mRNAs and lncRNAs according to the competing endogenous (ceRNA) hypothesis.^19^ Considering this hypothesis, we speculated that the 8 corresponding miRNAs of the down‐regulated CCL5 gene might be relatively highly expressed and that the 2 corresponding lncRNAs might expressed at low levels in antiviral therapy non‐responders. However, more laboratory experiments and clinical trials are needed to validate this hypothesis.

### 3.7 Verification of potential biomarker by measuring chemokine expression

**FIGURE 6:**
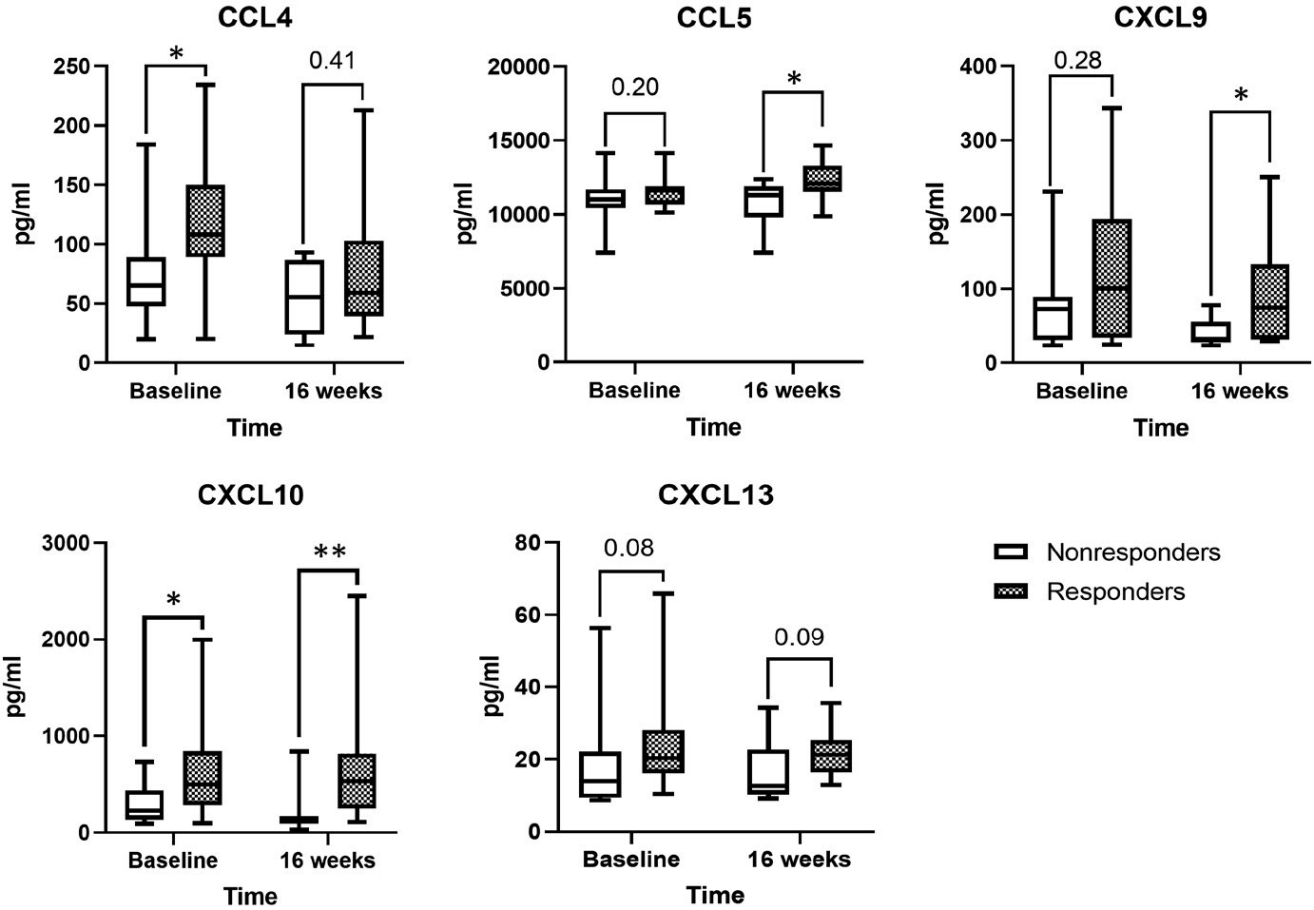
The expression level of CCL4, CCL5, CXCL9, CXCL10 and CXCL13 in serum of CHB therapeutic non‐responders were lower than that of responders at baseline and at 16 weeks of treatment. *P <.05, **P <.01

The hub genes were predicted to be mostly enriched in chemokine‐mediated signalling pathway. The expression levels of 5 hub chemokines were verified in CHB children serum samples using quantibody® array kit. There were no significant differences in age, weight, baseline viral load, alanine aminotransferase (ALT) levels or pathology between responders and non‐responders patients before treatment (Baseline). Demographic and clinical characteristics of the included CHB patients are shown in Table S6. Consistent with the prediction, the results showed that the expression levels of CCL4, CCL5, CXCL9, CXCL10 and CXCL13 in serum of non‐responders to antiviral treatment were generally lower than that of responders in the early phase of treatment (Figure 6 and Table S6). The levels of CCL4 and CXCL10 were significantly higher in responders than that of non‐responders at baseline (*P* < .05). Though the levels of CCL5 and CXCL9 were not significantly higher in responders than that of non‐responders at baseline, statistically differences were detected at 16 weeks of treatment (*P* < .05).

## 4 DISCUSSION

CHB still endangers human health, the outcome of which might be improved through antiviral therapy. However, the genetic differences between antiviral therapy responders and non‐responders remain unclear. Therefore, in this study we conducted bioinformatics analysis to identify a total of 1164 DEGs and 77 overlapping DEGs in CHB patients who received antiviral therapies. First, to systemically analyse the relationships and functions of overlapping DEGs, we constructed PPI networks. A variety of interactions among the selected DEGs was discovered, especially for the down‐regulated DEGs. As a result of PPI network analyses, 3 hub modules and 15 hub genes were discovered with the MCODE and cytoHubba algorithms in Cytoscape, respectively. Enrichment analyses of the selected DEGs, hub genes and hub modules all revealed that DEGs were mainly enriched in the immune response pathway, especially chemokine signalling pathways. We speculated that immune‐related pathways might be determinants of antiviral efficacy. Next, we conducted the immune cell infiltration analyses of the pre‐treated liver samples and detected significantly different characteristics between responders and non‐responders, indicating that the liver immunological microenvironment might affect the therapeutic responsiveness. Finally, to explore the potential molecular mechanism in the regulation of therapeutic responsiveness, upstream miRNAs and lncRNAs of hub genes were screened and a potential mRNA‐miRNA‐lncRNA triple‐regulatory network targeting CCL5 was established.

Intriguingly, many of the hub genes have been shown in previous studies to be involved in the progression or therapeutic responsiveness of CHB in previous studies, which partially supports the results of our bioinformatic analyses. First, among all the DEGs in our interaction network, CCL5 was the primary hub gene according to the five topological algorithms in the cytoHubba package. We also found that CCL5 is the only mRNA with validated interactions with miRNAs and lncRNAs in constructing the triple‐regulatory network. In a previous study, Hu et al found that CCL5 is a reliable biomarker for predicting liver fibrosis and cirrhosis: the expression of CCL5 in serum and hepatic tissue first increased in CHB patients with ongoing liver injury and then significantly decreased in advanced liver cirrhosis patients.^20^ Moreover, CCL5 and its receptor CCR5 have been established with roles in cancer progression and tumour immune evasion mechanisms, including in HCC.^21^, ^22^ An antagonist of CCL5 was found to ameliorate liver fibrosis and prevent HCC in mouse models, which further strengthened the relationship between CCL5 and liver fibrosis or carcinogenesis.^23^, ^24^ However, the effect of CCL5 on chronic liver disease progression and HCC development was reported to be more significant in steatohepatitis than in viral hepatitis.^25^ The changing levels of CCL5 (first increasing before decreasing) indicated that CCL5 might be regulated in multiple ways in CHB progression, a supposition that aligns with the complicated ncRNA regulatory network targeting CCL5 in our study. However, the regulatory mechanism remains unclear and deserves further research to explicit. Currently, the roles of CCL5 in HBV infection are difficult to reconcile and summarize. We found that CCL5 was significantly down‐regulated in non‐responders compared to responders before antiviral therapy. Low expression of CCL5 was potentially related to antiviral therapy failure, but further validation and exploration of the mechanism are needed. Second, the down‐regulated genes CXCL9, CXCL10 and CXCL13 detected by our studies were also emphasized and discussed in previous studies. In CHB patients who achieved HBsAg seroconversion under antiviral treatment, a decline in HBsAg was followed by the elevation of CXCL9, CXCL10, CXCL11, CXCL13 and IL‐21, suggesting the potential value of these chemokines in predicting a functional CHB cure.^26^ The level of serum CXCL9 before treatment was reported to be a good predictor of sustained virological response (SVR) in CHB patients receiving PEG‐IFN treatment, because it was significantly higher in patients with SVR than in those without SVR. The sensitivity of pre‐treatment CXCL9 was 59.1% in predicting patients with SVR, when the cut‐off value was set as >80 pg/mL.^27^ The levels of serum CXCL13, which is positively correlated with the levels of intrahepatic CXCL13 before treatment, were significantly higher in CHB patients with a complete response than in those without a complete response.^28^ In addition, serum levels of CXCL13 at the end of nucleos(t)ide analog(NA) treatment were positively associated with HBsAg loss and long‐term virological response.^29^ One of the potential mechanisms of CXCL13 against HBV infection was achieved by recruiting CXCR5+CD8+ T cells to the liver microenvironment and subsequently promoting the production of HBV‐specific IFNγ, IL‐21 and B cell antibody responses.^28^, ^30^ CXCR5, the chemokine receptor of CXCL13, was also closely associated with a lower clinical relapse rate upon cessation of the NA treatment in genetic variation studies of CHB patients.^31^ Based on previous studies and our findings, lower expression of pre‐treatment CXCL9, CXCL10 and CXCL13 as well as lower expression of post‐treatment CXCL13, probably predicts a better antiviral treatment response. Finally, STAT1, a key component in IFN signalling pathways and its upstream IFN/Janus kinase (JAK)/STAT signalling pathway, has also piqued interest in previous studies showing that HBV can influence the IFN/JAK/STAT signalling pathway and the differential phosphorylation of STAT1. Through STAT1‐related pathways, HBV infection not only induced monocytes to express lower levels of IFN signalling/stimulated genes and higher levels of IL‐10,^32^ but also led to the suppression of the anti‐HBV T cell response.^33^, ^34^ A specific enhancer of STAT1, 2‐NP, rescued IFN signalling in HBV‐infected monocytes,^32^ which corroborate our findings that a higher level of STAT1 is found in responders. More studies on the regulatory mechanism of IFN/JAK/STAT signalling may help elucidate the mechanism of persistent HBV infection and the poor response rates to antiviral therapy in CHB patients.

Immune response and chemokine signalling were revealed as the most significantly enriched in GO and KEGG pathways. Chemokines play various roles, including mediating immune cell trafficking, lymphoid tissue development and cancer progression by promoting the formation of an immunosuppressive microenvironment.^35^ In the present study, intrahepatic immune response activation and chemokine signalling were detected, which might result in different immune cell infiltration in the liver microenvironment between responders and non‐responders. In addition, in previous studies, the immune infiltration characteristics in the liver microenvironment were also reported to be associated with hepatitis severity, the HBV‐related liver disease progression and the efficacy of antiviral treatments in previous studies.^4^, ^36^ Our chemokine assays in serum of CHB patients confirmed that 5 hub chemokines (CCL4, CCL5, CXCL9, CXCL10 and CXCL13) were significantly down‐regulated in antiviral treatment non‐responders comparing non‐responders. The verified chemokines may have high potential values to be used as novel biomarkers in predicting treatment response.

Persistent HBV infection can cause immune suppression and dysfunction and induce HBV‐related liver disease. To reveal differences in immune cell infiltration characteristics between responders and non‐responders, we applied the CIBERSORT algorithm. Our results indicated a higher infiltration of CD8+ T cells and activated NK cells in non‐responders than in responders, which can be explained by the findings of previous studies. Progressive functional exhaustion and the ultimately deletion of virus‐specific T cells are commonly acknowledged in persistent HBV infection due to the production of inhibitory molecules such as PD‐1 and IL‐10 and the regulation of NK cells.^37^ In patients with poor control of their HBV infection control, many more CD8^+^ T cells infiltrated the livers, but the infiltration of HBV‐specific CD8^+^ T cells was attenuated by the recruitment of nonspecific CD8^+^ T cells into the liver microenvironment,^38^, ^39^ which promoted persistent HBV infection and liver pathogenesis.^40^ Activated NK cells played more pathogenic than protective roles in CHB, considering the preserved cytolytic activity, poor antiviral cytokine production and suppressive effect on HBV‐specific T cells.^41^ On the one hand, NK cells were activated and tended towards exhibiting cytolytic activity in the liver but without a concomitant increase in IFN‐γ production, which subsequently mediated infected hepatocyte injury but was insufficient for viral clearance; on the other hand, NK cells inhibited HBV‐specific T cell responses by affecting antigen‐presenting cells and regulating cytokines or killing T cells.^42^ PegIFNα and IFN‐stimulating TLR7 agonists therapy were reported to be efficient in restoring the function of NK cells and removing their negative effect on specific T cells.^43^, ^44^ However, the potential benefits of NK cell modulation in the setting of persistent HBV infection need to be further explored and demonstrated. In contrast, our results indicated that the levels of resting CD4^+^ memory T cells, plasma cells, γδT cells, resting dendritic cells and macrophages were significantly higher in responders than in non‐responders. Higher resting CD4 memory T cells were detected in responders, but no significant difference was detected in other subsets of CD4 T cells including naive and activated memory CD4 T cells. Compared to CD8^+^ T cell responses in CHB, much less is known about the down‐regulation of CD4^+^ T cell responses.^37^ The higher infiltration of plasma cells in responders might favour responsiveness to antiviral treatment. Although the relevance of B cells and the antibody response in CHB is not well understood, the antibody response to HBV may contribute to viral infection control by limiting viral spread and removing the circulating virions.^40^ γδT cells, a minor unique T cell subpopulation, have long been considered as innate‐like immune cells.^45^ In contrast to the elevated number and cytotoxic activity of γδT cells in chronic hepatitis C, the dominant subpopulation of γδT cells consisting of V_δ_2 T cells was significantly diminished in CHB and was accompanied by decreased IFN‐γ and cytotoxic activity.^46^ Our results revealed a lower level of γδT cells in non‐responders, indicating that the dysfunction of γδT cells might be more severe in this patient group. Dendritic cells (DCs), commonly considered as the most potent antigen‐presenting cells (APCs) that initiate primary immune responses, were reported to be lower in CHB patients than in healthy controls.^47^ Our studies revealed that the decline in resting DCs in non‐responders was more severe than that in responders (*P* <.05), possibly indicating a more severe dysfunction of DCs in non‐responders at baseline. However, the total levels of dendritic cells of responders and non‐responders were not significantly different prior to IFN‐α treatment (*P* = .16), which is consistent with previous findings of peripheral blood DCs in CHB patients.^48^, ^49^ The dysfunction of DCs may reduce exogenous IFN‐α secretion and might also lead to non‐responsiveness to IFN‐α treatment in CHB patients.^4^ However, recent studies investigating the frequency and functionality status of DCs in CHB infection are still insufficient. In addition to DCs, macrophages are also important components of APCs in HBV infection. Macrophages differentiate into various subsets and play divergent roles in interacting with HBV infection including pathogen clearance (mainly conducted by M1 macrophages) and pathogenesis (mainly conducted by M2 macrophages).^50^, ^51^ Conflicting findings on the roles of macrophage in CHB progression are difficult to reconcile and their roles in antiviral treatment remain unclear despite recent studies. Our results suggest a higher infiltration of macrophages in responders than non‐responders at baseline and an even higher infiltration of macrophages in responders after treatment than before treatment, which deserves to be further verified and explained in future experiments.

Increasing evidence indicates that ncRNAs, including miRNAs and lncRNAs are actively involved in various regulatory processes of HBV‐related liver diseases.^52^, ^53^, ^54^ miRNAs regulate gene expression by binding to target mRNAs, causing mRNA degradation or translation inhibition. lncRNAs have various functions in chromosome modification, transcriptional regulation and post‐transcriptional processing by interacting with multiple molecules including DNA, RNA and proteins. Based on the competing endogenous RNA (ceRNA) triple‐regulatory hypothesis, lncRNAs can competitively bind to miRNAs thereby relieving the suppressive effect of miRNAs on mRNAs.^19^ Recent studies have shown interest in revealing potential ceRNA interactions to better understand the molecular regulatory mechanisms in CHB. Fan et al revealed that the lncRNA n335586/miR‐924/CKMT1A axis can promote HBV‐related HCC cell migration and invasion.^55^ Liu et al found that the lncRNA H19/miR‐675/PPARα axis might regulate liver cell injury and energy metabolism remodelling induced by HBx via Akt/mTOR signalling.^56^ However, integrated and comprehensive analyses of the ceRNAs network in HBV infection have not sufficiently addressed these possibilities. In this study, we proposed a novel mRNA‐miRNA‐lncRNA triple‐regulatory network to explore potential ceRNA network interactions that may influence the process of CHB treatment. The subnetwork might provide novel therapeutic targets in hepatitis B and promising biomarkers for predicting the efficacy of antiviral therapy. However, notably, putative ceRNA interactions sometimes are not confirmed by experiments, because a single physiological ceRNA may have limited influence on highly expressed miRNAs.^57^ It is better to study ceRNAs collectively as a network instead of individually in the post‐transcription regulation processes. That is, putative ceRNA interactions should be scrutinized collectively using transcriptome‐wide approaches coupled with bioinformatic prediction.

In summary, antiviral therapeutic responsiveness is suboptimal and variable among chronic hepatitis B (CHB) patients, and the underlying mechanism is unclear. To find a valid regimen for treating CHB, we must know more about the host factors affecting therapeutic responsiveness. Our bioinformatics analysis revealed 5 hub chemokines (CCL4, CCL5, CXCL9, CXCL10 and CXCL13) as candidate biomarkers in predicting therapeutic responsiveness and a potential mRNA‐miRNA‐lncRNA as a potential regulatory network targeting CCL5. In addition, we found that the antiviral therapeutic responsiveness was associated with the host chemokine‐mediated immune response and immune cell infiltration characteristics in the liver microenvironment. However, the present study presented certain limitations. First, the major limitation of our study was the small sample numbers, which may influence the statistical power of our analyses. Due to the difficulty in completing the long follow‐up period, the number of included CHB patients in the present study was limited. We performed the chemokine assays to enlarge the sample sizes and verify some of our findings, but more clinical studies with larger sample sizes are needed to verify our findings in the future. Second, although our immune infiltration analyses lay a foundation for further researches, we could not validate the immune infiltration characteristics in CHB livers samples. Finally, the identified chemokines in blood samples may be valuable and applicable biomarkers in CHB treatment, but the molecular mechanism of the chemokine signalling pathways or the interactions between chemokines and the liver microenvironment needs to be further explored. Further studies considering these aspects should be conducted in the future.

## 5 CONCLUSION

In conclusion, we performed the first integrated bioinformatics analysis exploring the association between host genetic factors and antiviral therapeutic responsiveness in CHB patients. Our findings suggested that host genetic factors influenced therapeutic responsiveness in CHB patients, which may be related to the chemokine‐mediated immune response, liver immunological microenvironment and a potential mRNA‐miRNA‐lncRNA network targeting CCL5. Our study provides candidate biomarkers and potential molecular mechanisms of therapeutic responsiveness in CHB. However, more studies are needed to validate these findings.

## CONFLICT OF INTEREST

All the authors declare that there are no conflicts of interest relevant to this article.

## AUTHOR CONTRIBUTION

**Yi He:** Conceptualization (equal); Formal analysis (equal); Methodology (equal); Resources (equal); Software (equal); Validation (equal); Visualization (equal); Writing‐original draft (lead). **Yingzhi Zhou:** Funding acquisition (equal). **Huimin Wang:** Funding acquisition (equal). **Jingyang Yin:** Formal analysis (equal); Methodology (equal); Software (equal); Validation (equal); Writing‐original draft (supporting). **Yunan Chang:** Data curation (equal); Investigation (equal); Resources (equal); Software (equal); Validation (equal); Visualization (equal); Writing‐review & editing (equal). **Peng Hu:** Conceptualization (equal); Writing‐original draft (equal). **Hong Ren:** Conceptualization (equal); Writing‐original draft (equal). **Hongmei Xu:** Conceptualization (equal); Investigation (equal); Methodology (equal); Project administration (equal); Resources (equal); Supervision (equal); Validation (equal); Visualization (equal); Writing‐review & editing (lead).

## Supporting information

Fig S1

Table S1

Table S2

Table S3

Table S4

Table S5

Table S6

## Data Availability

Data sets used and analysed during the current study are available from the corresponding author on reasonable request.
