# Supplementary material for: Identifying potential biomarkers in hepatitis B virus infection and its response to the antiviral therapy by integrated bioinformatic analysis
Source: J Cell Mol Med. 2021 May 26;25(14):6558–72. doi: 10.1111/jcmm.16655 (PMC8278120; doi:10.1111/jcmm.16655)
Supplement: Supplementary file 1 — Fig S1 [file JCMM-25-6558-s001.pdf]

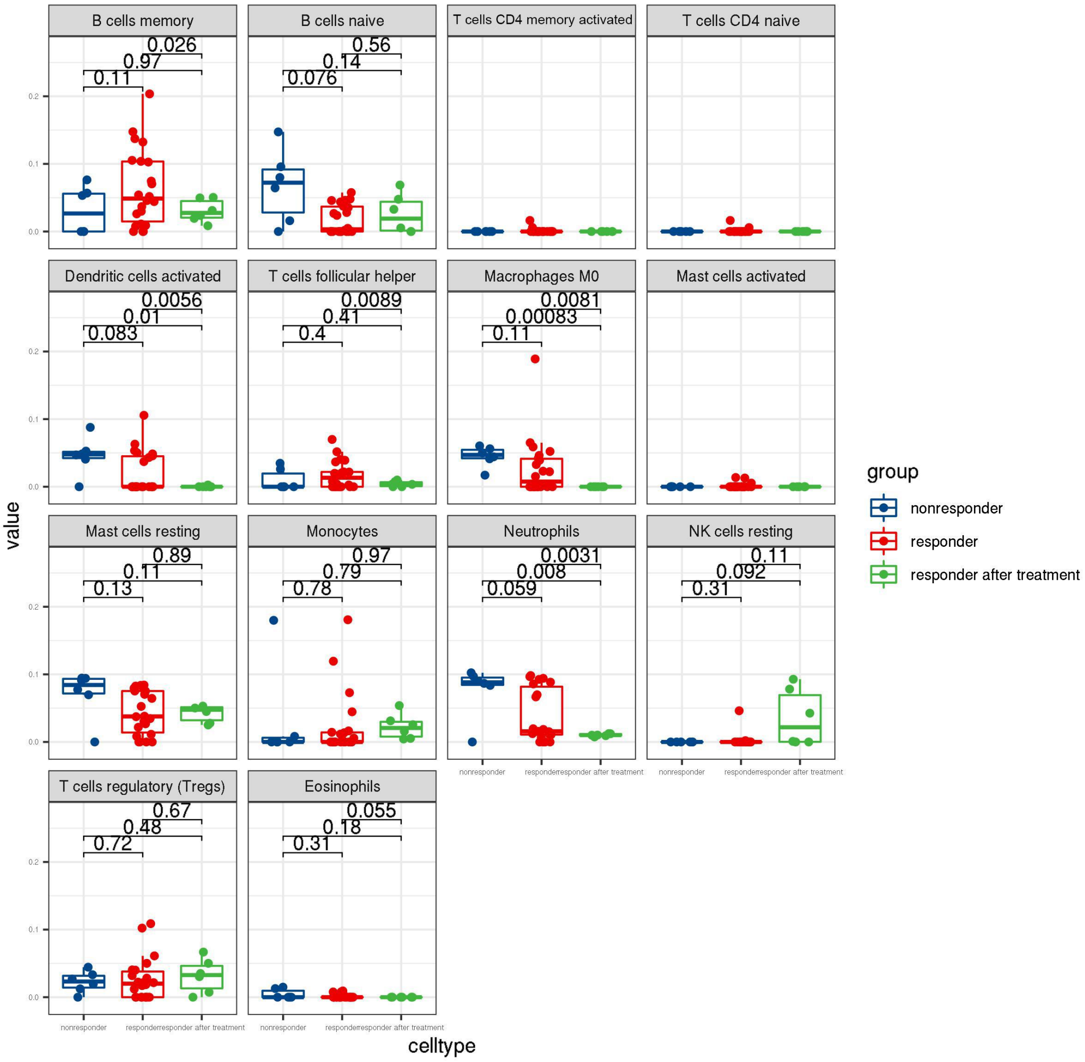

Figure S1. Immune cell infiltration characterization comparing responder and nonresponder. Blue, red and green color represent liver samples of responders before treatment, responders after treatment and nonresponders before treatment. Numbers above any two boxs represent *P*-value of student's T test(*P*-value <0.05 was considered to be significantly different).
