# Supplementary material for: Identifying potential biomarkers in hepatitis B virus infection and its response to the antiviral therapy by integrated bioinformatic analysis
Source: J Cell Mol Med. 2021 May 26;25(14):6558–72. doi: 10.1111/jcmm.16655 (PMC8278120; doi:10.1111/jcmm.16655)
Supplement: Supplementary file 2 — Table S1 [file JCMM-25-6558-s006.docx]

**Table S1. The selected 77 DEGs that are shared by ≥2 datasets.**

| Down-regulated DEGs | Up-regulated DEGs |
| --- | --- |
| SAMSN1  CXCL11  CXCL9  CXCL10  HKDC1  GPC3  UPP2  XIST  ASCL1  FCGR1B  CXCR4  C15orf48  CXCL13  GPNMB  AKR1B10  CHI3L1  HLA-DQB1  CCL20  SLAMF8  FCGR3A  CCL4  TYMS  UBD  LAMA3  S100A11  STAT1  UCP2  LGALS3  GBP1  CD74  FCGR3B  SLAMF7  CCL5  HLA-DMA  LYZ  IGSF6  APOL3  BIRC3  CTSC  MMS22L  HLA-DRB5  ANKRD22  HLA-DOA  DRAM1  TMPRSS3  MACC1  HLA-DRB1  ICOS  CD69  CRISPLD2  IGKV3-20  IGLJ3  IGHV3-30  H19  ENPP2  IGK  IGHV3-23  IGHA1 | MYBPC1  MYL2  PEG10  KLHL41  TNNC2  TNNC1  MYH1  MYH2  MYL1  MB  MT1F  SLN  KCNB1  ACTA1  CKM  SERPINA12  DHRS7C  TENM3  TTN |

DEGs：differential expressed genes
