# Supplementary material for: Identifying potential biomarkers in hepatitis B virus infection and its response to the antiviral therapy by integrated bioinformatic analysis
Source: J Cell Mol Med. 2021 May 26;25(14):6558–72. doi: 10.1111/jcmm.16655 (PMC8278120; doi:10.1111/jcmm.16655)
Supplement: Supplementary file 3 — Table S2 [file JCMM-25-6558-s002.docx]

**Table S2A. GO and KEGG pathway enrichment analysis of upregulated DEGs**

| Category | Term | Count | *P*-Value | Genes |
| --- | --- | --- | --- | --- |
| BP | GO:0030049~muscle filament sliding | 8 | 5.28E-15 | ACTA1,MYH2,MYBPC1,MYL1,MYL2,TNNC1,TNNC2,TTN |
|  | GO:0006936~muscle contraction | 5 | 4.45E-06 | ACTA1,MYH1,MYH2,MYL1,TTN |
|  | GO:0060048~cardiac muscle contraction | 4 | 1.43E-05 | MYL1,MYL2,TNNC1,TTN |
|  | GO:0030240~skeletal muscle thin filament assembly | 2 | 5.35E-03 | ACTA1,TTN |
| CC | GO:0005829~cytosol | 9 | 9.16E-03 | ACTA1,MYH2,MYBPC1,CKM,MYL1,MYL2,  TNNC1,TNNC2,TTN |
|  | GO:0005859~muscle myosin complex | 4 | 4.49E-07 | MYH1,MYH2,MYL1,TTN |
|  | GO:0030016~myofibril | 4 | 2.61E-06 | MYH2,MYBPC1,MYL1, MYL2 |
|  | GO:0030017~sarcomere | 4 | 7.23E-06 | ACTA1,MYH2,MYL1,MYL2 |
|  | GO:0031672~A band | 3 | 8.33E-05 | MYH1,MYH2,MYL2 |
|  | GO:0032982~myosin filament | 3 | 1.10E-04 | MYH1,MYH2,MYBPC1 |
|  | GO:0033017~sarcoplasmic reticulum membrane | 3 | 5.07E-04 | SLN, DHRS7C,KLHL41 |
|  | GO:0016459~myosin complex | 3 | 1.10E-03 | MYH1,MYH2,MYL2 |
|  | GO:0005861~troponin complex | 2 | 7.88E-03 | TNNC1,TNNC2 |
| MF | GO:0005509~calcium ion binding | 5 | 6.13E-03 | MYL1,MYL2,TNNC1,TNNC2, TTN |
|  | GO:0008307~structural constituent of muscle | 4 | 1.14E-05 | MYBPC1,MYL1,MYL2, TTN |
|  | GO:0003779~actin binding | 4 | 3.00E-03 | MYH1,MYH2,MYBPC1, TNNC2 |
|  | GO:0051015~actin filament binding | 3 | 8.55E-03 | TNNC1,TNNC2,TTN |
| KEEG | hsa05410:Hypertrophic cardiomyopathy(HCM) | 3 | 2.57E-03 | MYL2,TNNC1,TTN |
|  | hsa05414:Dilated cardiomyopathy | 3 | 2.97E-03 | MYL2,TNNC1,TTN |
|  | hsa04530:Tight junction | 3 | 3.19E-03 | MYH1,MYH2,MYL2 |

**Table S2B. GO and KEGG pathway enrichment analysis of down regulated DEGs**

| Category | Term | Count | *P*-Value | Genes |
| --- | --- | --- | --- | --- |
| BP | GO:0006955~immune response | 24 | 8.39E-24 | CD74,HLA-DRB5, CXCL9,IGSF6, IGHV3-30,CCL20,IGHV3-23,CXCL13,CXCL10, HLA-DMA,CXCL11,FCGR3A,FCGR3B,CCL5,CCL4,  ENPP2,IGHA1,HLA-DOA,ICOS,FCGR1B,IGKV3-20,  CTSC,HLA-DRB1,HLA-DQB1 |
|  | GO:0006954~inflammatory response | 11 | 1.71E-07 | CXCL10,CXCL11, CXCL9,CCL20,CCL5,CCL4,CXCR4, CHI3L1,CXCL13, LYZ, APOL3 |
|  | GO:0007165~signal transduction | 10 | 7.63E-03 | CXCL10,CD74,CXCL11, CXCL9,CCL20,CCL4,CD69,  HLA-DOA,APOL3,S100A11 |
|  | GO:0007186~G-protein coupled receptor signaling pathway | 9 | 5.37E-03 | CXCL10,CXCL11, CXCL9,CCL20,CCL5,CCL4,ENPP2,  CXCR4,CXCL13 |
|  | GO:0070098~chemokine-mediated signaling pathway | 8 | 1.78E-09 | CXCL10,CXCL11, CXCL9,CCL20,CCL5,CCL4,CXCR4,  CXCL13 |
|  | GO:0006935~chemotaxis | 7 | 1.79E-06 | CXCL10,CXCL11, CXCL9,CCL20,CCL5,ENPP2,CXCR4 |
|  | GO:0006898~receptor-mediated endocytosis | 7 | 2.03E-05 | IGHV3-30,TMPRSS3,IGHV3-23,ENPP2,IGHA1,  FCGR1B,IGKV3-20 |
|  | GO:0007267~cell-cell signaling | 7 | 1.15E-04 | CXCL10,CXCL11, CXCL9,CCL20,CCL5,CCL4,CXCL13 |
|  | GO:0060333~interferon-gamma-mediated signaling pathway | 6 | 2.37E-06 | HLA-DRB5, STAT1,FCGR1B, GBP1,HLA-DRB1,  HLA-DQB1 |
|  | GO:0019886~antigen process and presentation of exogenous peptide antigen via MHC class II | 6 | 8.52E-06 | CD74,HLA-DMA, HLA-DRB5, HLA-DOA, HLA-DRB1,  HLA-DQB1 |
|  | GO:0070374~positive regulation of ERK1 and ERK2 cascade | 6 | 1.85E-04 | CD74,GPNMB,CCL20,CCL5,CCL4,CHI3L1 |
|  | GO:0002504~antigen process and presentation of peptide or polysaccharide antigen via MHC class II | 5 | 1.74E-07 | HLA-DMA,HLA-DRB5,HLA-DOA,HLA-DRB1,  HLA-DQB1 |
|  | GO:0050776~regulation of immune response | 5 | 2.07E-03 | FCGR3A,IGHV3-30,IGHV3-23,SLAMF7,IGKV3-20 |
|  | GO:0042127~regulation of cell proliferation | 5 | 2.38E-03 | CXCL10,CXCL11, CXCL9,CXCL13,S100A11 |
|  | GO:0002548~monocyte chemotaxis | 4 | 2.79E-04 | LGALS3,CCL20,CCL5,CCL4 |
|  | GO:0019882~antigen process and presentation | 4 | 6.19E-04 | CD74,HLA-DRB5, HLA-DRB1, HLA-DQB1 |
|  | GO:0060326~cell chemotaxis | 4 | 1.01E-03 | CXCL10,CXCL9,CCL20,CCL5 |
|  | GO:0030593~neutrophil chemotaxis | 4 | 1.06E-03 | LGALS3,CCL20,CCL5,CCL4 |
|  | GO:0071347~cellular response to interleukin-1 | 4 | 1.30E-03 | CCL20,CCL5,CCL4,CHI3L1 |
|  | GO:0031295~T cell costimulation | 4 | 1.71E-03 | HLA-DRB5, ICOS, HLA-DRB1, HLA-DQB1 |
|  | GO:0006958~complement activation,classical pathway | 4 | 3.37E-03 | IGHV3-30,IGHV3-23,IGHA1,IGKV3-20 |
|  | GO:0071356~cellular response to tumor necrosis factor | 4 | 4.53E-03 | CCL20,CCL5,CCL4,CHI3L1 |
|  | GO:0038096~Fc-gamma receptor signaling pathway involved in phagocytosis | 4 | 6.75E-03 | FCGR3A,IGHV3-30,IGHV3-23,IGKV3-20 |
|  | GO:0002381~immunoglobulin production involved in immunoglobulin mediated immune response | 3 | 8.99E-05 | HLA-DRB5, HLA-DRB1, HLA-DQB1 |
|  | GO:0030816~positive regulation of cAMP metobolic process | 3 | 1.35E-04 | CXCL10,CXCL11, CXCL9 |
|  | GO:0002455~humoral immune response mediated by circulating immunoglobulin | 3 | 1.88E-04 | HLA-DRB5, HLA-DRB1, HLA-DQB1 |
|  | GO:2000406~positive regulation of T cell migration | 3 | 4.01E-04 | CXCL10,CCL20,CCL5 |
|  | GO:0043950~positive regulation of cAMP-mediated signaling | 3 | 5.85E-04 | CXCL10,CXCL11, CXCL9 |
|  | GO:0002690~positive regulation of leukocyte chemotaxis | 3 | 1.34E-03 | CXCL10,CXCL11, CXCL9 |
|  | GO:0051281~positive regulation of release of sequestered calcium ion into cytosol | 3 | 3.02E-03 | CXCL10,CXCL11, CXCL9 |
|  | GO:0045765~regulation of angiogenesis | 3 | 3.98E-03 | GPNMB,ENPP2,CXCL13 |
|  | GO:0050918~positive chemotaxis | 3 | 5.05E-03 | LGALS3,GPNMB, CCL5 |
|  | GO:0042130~negative regulation of T cell proliferation | 3 | 5.63E-03 | HLA-DRB5, GPNMB,HLA-DRB1 |
|  | GO:0051262~protein tetramerization | 3 | 6.55E-03 | HLA-DRB5, CCL5,HLA-DRB1 |
|  | GO:2001179~regulation of interleukin-10 secretion | 2 | 9.08E-03 | HLA-DRB5, HLA-DRB1 |
| CC | GO:0005615~extracellular space | 19 | 1.47E-08 | HLA-DRB5, CXCL9,IGHV3-30,CCL20,IGHV3-23,  CXCL13,LYZ,CXCL10,LGALS3,CXCL11,CCL5,CCL4,  ENPP2,GPC3,CHI3L1,IGHA1,S100A11,IGKV3-20,CTSC |
|  | GO:0070062~extracellular exoxome | 19 | 5.01E-04 | CD74,HLA-DRB5, LAMA3,IGHV3-23,CXCR4,LYZ,  LGALS3,HLA-DMA,FCGR3A, FCGR3B,AKR1B10,  CRISPLD2,GPC3,CHI3L1,IGHA1,S100A11,IGKV3-20,  CTSC,HLA-DRB1 |
|  | GO:0005576~extracellular region | 17 | 6.13E-06 | CXCL9,IGHV3-30,CCL20,LAMA3,IGHV3-23,CXCL13,LYZ,CXCL10,CXCL11,CRISPLD2,CCL5,CCL4,IGHA1,  ICOS, GBP1,APOL3,IGKV3-20 |
|  | GO:0009897~external side of plasma membrane | 10 | 8.18E-09 | CXCL10,CD74,FCGR3A,HLA-DRB5,CXCL9,IGHV3-23,CD69,IGHA1,ICOS,HLA-DRB1 |
|  | GO:0005765~lysosomal membrane | 7 | 1.25E-04 | CD74,HLA-DMA, HLA-DRB5, DRAM1,HLA-DOA,  HLA-DRB1, HLA-DQB1 |
|  | GO:0042613~MHC class II protein complex | 6 | 3.94E-09 | CD74,HLA-DMA, HLA-DRB5, HLA-DOA, HLA-DRB1,  HLA-DQB1 |
|  | GO:0030669~clathrin-coated endocytic vesicle membrane | 5 | 5.52E-06 | CD74,HLA-DRB5, FCGR1B,HLA-DRB1,HLA-DQB1 |
|  | GO:0071556~integral component of lumenal side of endoplasmic reticulum membrane | 4 | 7.60E-05 | CD74,HLA-DRB5, HLA-DRB1, HLA-DQB1 |
|  | GO:0030658~transport vesical membrane | 4 | 1.72E-04 | CD74,HLA-DRB5, HLA-DRB1, HLA-DQB1 |
|  | GO:0012507~ER to Golgi transport vesical membrane | 4 | 4.39E-04 | CD74,HLA-DRB5, HLA-DRB1, HLA-DQB1 |
|  | GO:0030666~endocytic vesical membrane | 4 | 8.83E-04 | CD74,HLA-DRB5, HLA-DRB1, HLA-DQB1 |
|  | GO:0032588~trans-Golgi network membrane | 4 | 1.71E-03 | CD74,HLA-DRB5, HLA-DRB1, HLA-DQB1 |
| MF | GO:0008009~chemokine activity | 7 | 5.54E-09 | CXCL10,CXCL11, CXCL9,CCL20,CCL5,CCL4,CXCL13 |
|  | GO:0008201~heparin binding | 5 | 1.18E-03 | CXCL10,CXCL11, GPNMB,CRISPLD2,CXCL13 |
|  | GO:0004252~serine-type endopeptidase activity | 5 | 6.33E-03 | IGHV3-30,TMPRSS3,IGHV3-23,IGKV3-20, CTSC |
|  | GO:0048248~CXCR3 chemokin receptor binding | 4 | 2.29E-07 | CXCL10,CXCL11, CXCL9,CXCL13 |
|  | GO:0032395~MHC class II receptor activity | 4 | 1.02E-05 | HLA-DMA,HLA-DOA,HLA-DRB1,HLA-DQB1 |
|  | GO:0023026~MHC class II protein complex bingding | 4 | 1.25E-05 | CD74,HLA-DMA,HLA-DOA,HLA-DRB1 |
|  | GO:0003823~antigen bingding | 4 | 3.32E-03 | IGHV3-30,IGHV3-23,IGHA1,IGKV3-20 |
|  | GO:0031730~CCR5 chemokin receptor binding | 3 | 2.29E-04 | STAT1,CCL5,CCL4 |
|  | GO:0019864~IgG binding | 3 | 4.46E-04 | FCGR3A,FCGR3B,FCGR1B |
|  | GO:0019955~cytokine binding | 3 | 1.37E-03 | CD74,CXCR4,GBP1 |
|  | GO:0042056~chemoattractant activity | 3 | 2.77E-03 | LGALS3,GPNMB, CCL5 |
|  | GO:0042605~peptide antigen binding | 3 | 2.97E-03 | HLA-DRB5, HLA-DRB1, HLA-DQB1 |
| KEEG | hsa04062:Chemokine signaling pathway | 9 | 3.51E-07 | CXCL10,CXCL11, CXCL9,STAT1,CCL20,CCL5,CCL4,  CXCR4,CXCL13 |
|  | hsa05152:Tuberculosis | 9 | 2.40E-07 | CD74,HLA-DMA, FCGR3A, HLA-DRB5, FCGR3B,  STAT1,HLA-DOA,HLA-DRB1,HLA-DQB1 |
|  | hsa04060:Cytokine-cytokine receptor interaction | 8 | 3.05E-05 | CXCL10,CXCL11, CXCL9,CCL20,CCL5,CCL4,CXCR4,  CXCL13 |
|  | hsa05140:Leishmaniasis | 8 | 6.95E-09 | HLA-DMA,FCGR3A,HLA-DRB5,FCGR3B, STAT1,  HLA-DOA,HLA-DRB1,HLA-DQB1 |
|  | hsa05164:Influenza A | 8 | 3.40E-06 | CXCL10,HLA-DMA,HLA-DRB5,STAT1,CCL5,  HLA-DOA,HLA-DRB1,HLA-DQB1 |
|  | hsa05168:Herpes simplex infection | 8 | 4.76E-06 | CD74,HLA-DMA,HLA-DRB5,STAT1,CCL5,HLA-DOA,HLA-DRB1, HLA-DQB1 |
|  | hsa05145:Toxoplasmosis | 8 | 1.51E-07 | HLA-DMA,HLA-DRB5,STAT1,LAMA3,HLA-DOA,  HLA-DRB1, BIRC3,HLA-DQB1 |
|  | hsa04672:Intestinal immune network for IgA production | 7 | 1.94E-08 | HLA-DMA, HLA-DRB5, CXCR4,HLA-DOA,ICOS,  HLA-DRB1, HLA-DQB1 |
|  | hsa05150:Staphylococcus aureus infection | 7 | 4.59E-08 | HLA-DMA, FCGR3A,HLA-DRB5,FCGR3B,  HLA-DOA, HLA-DRB1, HLA-DQB1 |
|  | hsa05323:Rheumatoid arthritis | 7 | 8.80E-07 | HLA-DMA, HLA-DRB5, CCL20, CCL5,  HLA-DOA, HLA-DRB1, HLA-DQB1 |
|  | hsa05322:Systemic lupus erythematosus | 7 | 1.03E-05 | HLA-DMA, FCGR3A, HLA-DRB5, FCGR3B,  HLA-DOA, HLA-DRB1, HLA-DQB1 |
|  | hsa04145:Phagosome | 7 | 1.97E-05 | HLA-DMA, FCGR3A, HLA-DRB5, FCGR3B,  HLA-DOA, HLA-DRB1, HLA-DQB1 |
|  | hsa05321:Inflammatory bowel disease(IBD) | 6 | 4.10E-06 | HLA-DMA, HLA-DRB5, STAT1, HLA-DOA,  HLA-DRB1, HLA-DQB1 |
|  | hsa04612:Antigen process and presentation | 6 | 9.62E-06 | CD74,HLA-DMA, HLA-DRB5, HLA-DOA, HLA-DRB1,  HLA-DQB1 |
|  | hsa04620:Toll-like receptor sinaling pathway | 6 | 4.87E-05 | CXCL10,CXCL11, CXCL9,STAT1,CCL5,CCL4 |
|  | hsa04514:Cell adhesion molecules(CAMs) | 6 | 1.96E-04 | HLA-DMA, HLA-DRB5, HLA-DOA, ICOS, HLA-DRB1,  HLA-DQB1 |
|  | hsa05310:Asthma | 5 | 4.81E-06 | HLA-DMA, HLA-DRB5, HLA-DOA, HLA-DRB1,  HLA-DQB1 |
|  | hsa05332:Graft-versus-host disease | 5 | 7.13E-06 | HLA-DMA, HLA-DRB5, HLA-DOA, HLA-DRB1,  HLA-DQB1 |
|  | hsa05330:Allograft rejection | 5 | 1.14E-05 | HLA-DMA, HLA-DRB5, HLA-DOA, HLA-DRB1,  HLA-DQB1 |
|  | hsa04940:Type I diabetes mellitus | 5 | 1.90E-05 | HLA-DMA, HLA-DRB5, HLA-DOA, HLA-DRB1,  HLA-DQB1 |
|  | hsa05320:Autoimmune thyroid disease | 5 | 4.48E-05 | HLA-DMA, HLA-DRB5, HLA-DOA, HLA-DRB1,  HLA-DQB1 |
|  | hsa05416:Viral myocarditis | 5 | 6.45E-05 | HLA-DMA, HLA-DRB5, HLA-DOA, HLA-DRB1,  HLA-DQB1 |
|  | hsa04668:TNF sinaling pathway | 4 | 8.16E-03 | CXCL10,CCL20,CCL5,BIRC3 |

GO:Gene Ontology; KEGG:Kyoto Encyclopedia of Genes and Genomes; DEGs：differential expressed genes; BP:biological process; CC:cellular component; MF: molecular function.
