# Supplementary material for: Identifying potential biomarkers in hepatitis B virus infection and its response to the antiviral therapy by integrated bioinformatic analysis
Source: J Cell Mol Med. 2021 May 26;25(14):6558–72. doi: 10.1111/jcmm.16655 (PMC8278120; doi:10.1111/jcmm.16655)
Supplement: Supplementary file 4 — Table S3 [file JCMM-25-6558-s007.docx]

**Table S3. The top 25 genes ranked in cytoHubba by five classification methods.**

| Degree | Stress | MNC | Radiality | Closeness |
| --- | --- | --- | --- | --- |
| CXCL9 | FCGR3A | CCL5 | CCL5 | CCL5 |
| CCL5 | MYH2 | CXCL9 | FCGR3A | CD74 |
| CXCR4 | LGALS3 | TTN | CD74 | CXCL9 |
| TTN | CD74 | MYL1 | LGALS3 | FCGR3A |
| ACTA1 | CCL5 | CXCR4 | HLA-DRB1 | CXCR4 |
| MYH2 | ACTA1 | MYL2 | CCL4 | CCL4 |
| MYL1 | GBP1 | MYH2 | CXCL9 | HLA-DRB1 |
| CD74 | CXCL9 | CXCL10 | CXCR4 | MYH2 |
| GBP1 | CTSC | MYH1 | MYH2 | CD69 |
| CCL4 | HLA-DRB1 | STAT1 | GBP1 | GBP1 |
| MYL2 | CD69 | CCL4 | CD69 | CXCL10 |
| CD69 | CCL4 | ACTA1 | FCGR1B | ACTA1 |
| CXCL10 | CXCR4 | TNNC2 | HLA-DRB5 | LGALS3 |
| MYH1 | HLA-DRB5 | CXCL13 | CXCL10 | HLA-DQB1 |
| HLA-DQB1 | HLA-DQB1 | HLA-DRB1 | HLA-DQB1 | HLA-DRB5 |
| HLA-DRB5 | FCGR1B | CXCL11 | STAT1 | STAT1 |
| STAT1 | SLAMF8 | HLA-DQB1 | ACTA1 | CXCL11 |
| TNNC2 | CXCL13 | HLA-DRB5 | CXCL11 | CXCL13 |
| CXCL13 | GPNMB | MYBPC1 | SLAMF8 | FCGR1B |
| HLA-DRB1 | TTN | TNNC1 | CXCL13 | CCL20 |
| CXCL11 | MYL1 | CD69 | CCL20 | TTN |
| MYBPC1 | CHI3L1 | CKM | GPNMB | MYL1 |
| TNNC1 | CKM | CCL20 | CKM | MYL2 |
| FCGR3A | CXCL10 | CD74 | HLA-DMA | MYH1 |
| CKM | STAT1 | FCGR1B | HLA-DOA | CKM |
