# Supplementary material for: Identifying potential biomarkers in hepatitis B virus infection and its response to the antiviral therapy by integrated bioinformatic analysis
Source: J Cell Mol Med. 2021 May 26;25(14):6558–72. doi: 10.1111/jcmm.16655 (PMC8278120; doi:10.1111/jcmm.16655)
Supplement: Supplementary file 5 — Table S4 [file JCMM-25-6558-s003.docx]

**Table S4. The immune cell composition for each sample predicted by Cibersort. Numbers in the box represent the proportions of the immnune cells in each sample of the 3 datasets GSE27555,GSE54747 and GSE66698.**

| **Datasets** | **Sample** | **Bcells naive** | **Bcells memory** | **Plasma**  **cells** | **T cells CD8** | **T cells CD4 naive** | **T cells CD4**  **memory**  **resting** | **T cells CD4**  **memory**  **activated** | **T cells**  **follicular**  **helper** | **T cells**  **regulatory**  **(Tregs)** | **T cells**  **gamma**  **delta** | **NK cells**  **resting** | **NK cells**  **activated** | **Monocytes** | **Macrophages**  **M0** | **Macrophages**  **M1** | **Macrophages**  **M2** | **Dendritic**  **cells**  **resting** | **Dendritic**  **cells**  **activated** | **Mast cells**  **resting** | **Mast cells**  **activated** | **Eosinophils** | **Neutrophils** | **P-value** | **Pearson Correlation** |
| --- | --- | --- | --- | --- | --- | --- | --- | --- | --- | --- | --- | --- | --- | --- | --- | --- | --- | --- | --- | --- | --- | --- | --- | --- | --- |
| **GSE27555** | **GSM680443** | **0.113** | **0** | **0.117** | **0.108** | **0** | **0.173** | **0** | **0** | **0.014** | **0** | **0.011** | **0.033** | **0.13** | **0** | **0.048** | **0.25** | **0** | **0** | **0** | **0.004** | **0** | **0** | **0.640** | **0.005** |
|  | **GSM680444** | **0.08** | **0** | **0.096** | **0.22** | **0** | **0.023** | **0** | **0** | **0.019** | **0** | **0** | **0.047** | **0.18** | **0.06** | **0.123** | **0.151** | **0** | **0** | **0** | **0** | **0** | **0** | **0.430** | **0.030** |
|  | **GSM680445** | **0.082** | **0** | **0.104** | **0.071** | **0.051** | **0.139** | **0** | **0.018** | **0.075** | **0** | **0** | **0.143** | **0.031** | **0** | **0.211** | **0.038** | **0.014** | **0** | **0** | **0.02** | **0.003** | **0** | **0.590** | **0.011** |
|  | **GSM680446** | **0.093** | **0** | **0.097** | **0.142** | **0.017** | **0.234** | **0** | **0** | **0.03** | **0** | **0** | **0.08** | **0.116** | **0.026** | **0.063** | **0.072** | **0** | **0** | **0.03** | **0** | **0** | **0** | **0.700** | **-0.005** |
|  | **GSM680447** | **0.005** | **0.008** | **0.062** | **0.075** | **0** | **0.032** | **0.016** | **0.008** | **0.028** | **0.017** | **0** | **0.085** | **0.181** | **0.189** | **0.165** | **0.104** | **0** | **0** | **0.027** | **0** | **0** | **0** | **0.030** | **0.170** |
|  | **GSM680448** | **0.08** | **0** | **0.031** | **0.134** | **0** | **0.184** | **0** | **0** | **0.041** | **0** | **0.068** | **0.036** | **0.213** | **0** | **0.053** | **0.156** | **0** | **0** | **0** | **0.005** | **0** | **0** | **0.680** | **-0.002** |
|  | **GSM680449** | **0.032** | **0** | **0.077** | **0.158** | **0** | **0.207** | **0** | **0** | **0** | **0** | **0.035** | **0.039** | **0.181** | **0** | **0.096** | **0.174** | **0** | **0** | **0** | **0.001** | **0** | **0** | **0.500** | **0.020** |
|  | **GSM680450** | **0.048** | **0** | **0.209** | **0.235** | **0.052** | **0.053** | **0** | **0** | **0** | **0.025** | **0** | **0.043** | **0.073** | **0.022** | **0.075** | **0.164** | **0** | **0** | **0** | **0** | **0** | **0** | **0.250** | **0.048** |
|  | **GSM680451** | **0.025** | **0** | **0.006** | **0.33** | **0** | **0.033** | **0** | **0.03** | **0.039** | **0** | **0** | **0.164** | **0.105** | **0** | **0.081** | **0.136** | **0** | **0** | **0** | **0.033** | **0** | **0.017** | **0.500** | **0.020** |
|  | **GSM680452** | **0.046** | **0** | **0.11** | **0.317** | **0** | **0.08** | **0** | **0** | **0** | **0** | **0** | **0.092** | **0.119** | **0.023** | **0.141** | **0.067** | **0** | **0** | **0** | **0.005** | **0** | **0** | **0.440** | **0.030** |
|  | **GSM680453** | **0.121** | **0** | **0.138** | **0.067** | **0.057** | **0.125** | **0** | **0.006** | **0.039** | **0** | **0.028** | **0.042** | **0.121** | **0.039** | **0.096** | **0.078** | **0.007** | **0** | **0** | **0.012** | **0.025** | **0** | **0.640** | **0.006** |
|  | **GSM680454** | **0.102** | **0** | **0.09** | **0.209** | **0** | **0.182** | **0** | **0** | **0.004** | **0** | **0** | **0.06** | **0.128** | **0.003** | **0.073** | **0.131** | **0** | **0** | **0.019** | **0** | **0** | **0** | **0.590** | **0.011** |
|  | **GSM680455** | **0.099** | **0** | **0.079** | **0** | **0.185** | **0.075** | **0** | **0.017** | **0.046** | **0** | **0** | **0.119** | **0.081** | **0** | **0.047** | **0.225** | **0.004** | **0** | **0** | **0.004** | **0.018** | **0** | **0.750** | **-0.009** |
| **GSE54747** | **GSM1323157** | **0.064** | **0.053** | **0** | **0.341** | **0** | **0** | **0** | **0.035** | **0** | **0** | **0** | **0.178** | **0** | **0.044** | **0** | **0** | **0** | **0.088** | **0.094** | **0** | **0** | **0.102** | **0.470** | **0.036** |
|  | **GSM1323158** | **0.147** | **0** | **0** | **0.445** | **0** | **0** | **0** | **0.026** | **0.012** | **0** | **0** | **0.142** | **0** | **0.017** | **0** | **0** | **0.001** | **0.041** | **0.07** | **0** | **0.013** | **0.087** | **0.480** | **0.035** |
|  | **GSM1323159** | **0.051** | **0.06** | **0** | **0.377** | **0** | **0** | **0** | **0.028** | **0** | **0** | **0** | **0.186** | **0** | **0.056** | **0** | **0** | **0** | **0.041** | **0.092** | **0** | **0.017** | **0.093** | **0.500** | **0.033** |
|  | **GSM1323160** | **0** | **0.204** | **0** | **0.312** | **0** | **0** | **0** | **0.019** | **0.022** | **0** | **0** | **0.175** | **0** | **0.065** | **0** | **0** | **0** | **0.05** | **0.083** | **0** | **0** | **0.07** | **0.380** | **0.044** |
|  | **GSM1323161** | **0** | **0.137** | **0** | **0.391** | **0** | **0** | **0** | **0.022** | **0** | **0** | **0** | **0.202** | **0** | **0.052** | **0** | **0** | **0** | **0.043** | **0.079** | **0** | **0.008** | **0.067** | **0.490** | **0.034** |
|  | **GSM1323162** | **0** | **0.159** | **0** | **0.369** | **0** | **0** | **0** | **0.06** | **0** | **0** | **0** | **0.177** | **0** | **0.03** | **0** | **0** | **0** | **0.038** | **0.087** | **0** | **0.009** | **0.073** | **0.520** | **0.032** |
|  | **GSM1323163** | **0.044** | **0.046** | **0** | **0.435** | **0** | **0** | **0** | **0.04** | **0** | **0** | **0** | **0.178** | **0** | **0.033** | **0** | **0** | **0** | **0.037** | **0.08** | **0** | **0.009** | **0.098** | **0.470** | **0.036** |
|  | **GSM1323164** | **0** | **0.132** | **0** | **0.351** | **0** | **0** | **0** | **0.052** | **0** | **0** | **0** | **0.19** | **0** | **0.042** | **0** | **0** | **0** | **0.063** | **0.084** | **0** | **0** | **0.086** | **0.410** | **0.041** |
|  | **GSM1323165** | **0.096** | **0** | **0** | **0.454** | **0** | **0** | **0** | **0** | **0.033** | **0** | **0** | **0.143** | **0** | **0.041** | **0** | **0** | **0** | **0.049** | **0.094** | **0** | **0** | **0.09** | **0.360** | **0.045** |
|  | **GSM1323166** | **0** | **0.076** | **0** | **0.438** | **0** | **0** | **0** | **0** | **0.027** | **0** | **0** | **0.152** | **0.008** | **0.056** | **0** | **0** | **0** | **0.053** | **0.091** | **0** | **0.015** | **0.084** | **0.380** | **0.045** |
|  | **GSM1323167** | **0.016** | **0.057** | **0** | **0.447** | **0** | **0** | **0** | **0** | **0.044** | **0** | **0** | **0.165** | **0** | **0.05** | **0** | **0** | **0** | **0.047** | **0.077** | **0** | **0** | **0.097** | **0.320** | **0.059** |
|  | **GSM1323168** | **0** | **0.148** | **0** | **0.384** | **0** | **0** | **0** | **0.07** | **0** | **0** | **0** | **0.168** | **0** | **0.015** | **0** | **0** | **0** | **0.046** | **0.07** | **0** | **0.003** | **0.097** | **0.350** | **0.049** |
|  | **GSM1323169** | **0.035** | **0.052** | **0** | **0.391** | **0** | **0** | **0** | **0.019** | **0.017** | **0** | **0** | **0.144** | **0** | **0.059** | **0** | **0** | **0** | **0.106** | **0.084** | **0** | **0** | **0.092** | **0.340** | **0.053** |
|  | **GSM1323170** | **0** | **0.105** | **0** | **0.431** | **0** | **0** | **0** | **0.022** | **0.012** | **0** | **0** | **0.159** | **0** | **0.046** | **0** | **0** | **0** | **0.053** | **0.075** | **0** | **0.008** | **0.088** | **0.340** | **0.054** |
|  | **GSM1323171** | **0.027** | **0.075** | **0** | **0.405** | **0** | **0** | **0** | **0.039** | **0** | **0** | **0** | **0.197** | **0** | **0.039** | **0** | **0** | **0** | **0.049** | **0.075** | **0** | **0** | **0.094** | **0.470** | **0.038** |
| **GSE66698** | **GSM1629354** | **0.024** | **0.009** | **0.083** | **0.163** | **0** | **0.154** | **0** | **0.021** | **0.05** | **0.101** | **0** | **0** | **0** | **0** | **0.115** | **0.259** | **0** | **0** | **0.011** | **0** | **0** | **0.011** | **0.090** | **0.139** |
|  | **GSM1629355** | **0** | **0.044** | **0.195** | **0.06** | **0** | **0.147** | **0** | **0** | **0.019** | **0.099** | **0** | **0.046** | **0** | **0** | **0.085** | **0.264** | **0.013** | **0** | **0** | **0.013** | **0** | **0.015** | **0.050** | **0.224** |
|  | **GSM1629356** | **0.046** | **0.026** | **0.125** | **0.156** | **0** | **0.111** | **0** | **0.02** | **0.022** | **0.004** | **0** | **0.029** | **0.014** | **0** | **0.138** | **0.215** | **0.015** | **0** | **0.064** | **0** | **0** | **0.015** | **0.050** | **0.182** |
|  | **GSM1629357** | **0** | **0.07** | **0.157** | **0.141** | **0** | **0.179** | **0** | **0** | **0** | **0.039** | **0** | **0.044** | **0.006** | **0** | **0.14** | **0.159** | **0.011** | **0** | **0.038** | **0** | **0** | **0.016** | **0.030** | **0.260** |
|  | **GSM1629358** | **0.058** | **0** | **0.155** | **0.139** | **0** | **0.16** | **0** | **0** | **0.109** | **0** | **0** | **0** | **0.016** | **0** | **0.071** | **0.26** | **0.01** | **0** | **0.022** | **0** | **0** | **0** | **0.200** | **0.065** |
|  | **GSM1629359** | **0** | **0.104** | **0.067** | **0** | **0** | **0.204** | **0** | **0.003** | **0.032** | **0.111** | **0** | **0.02** | **0** | **0** | **0.118** | **0.269** | **0.016** | **0** | **0.037** | **0** | **0** | **0.019** | **0.090** | **0.128** |
|  | **GSM1629360** | **0** | **0.103** | **0.175** | **0.022** | **0** | **0.114** | **0** | **0** | **0.061** | **0.037** | **0.002** | **0.005** | **0.014** | **0** | **0.119** | **0.31** | **0.013** | **0** | **0** | **0.014** | **0** | **0.011** | **0.050** | **0.205** |
|  | **GSM1629361** | **0.028** | **0.037** | **0.06** | **0.071** | **0** | **0.193** | **0** | **0** | **0.102** | **0** | **0.046** | **0.006** | **0.045** | **0** | **0.081** | **0.271** | **0.009** | **0** | **0.035** | **0** | **0** | **0.016** | **0.410** | **0.032** |
|  | **GSM1629362** | **0.069** | **0.009** | **0.101** | **0.043** | **0** | **0.169** | **0** | **0.004** | **0.035** | **0** | **0.078** | **0** | **0.031** | **0** | **0.061** | **0.333** | **0.009** | **0** | **0.045** | **0** | **0** | **0.012** | **0.250** | **0.056** |
|  | **GSM1629363** | **0.005** | **0.019** | **0.101** | **0.005** | **0** | **0.302** | **0** | **0.008** | **0.007** | **0.048** | **0** | **0.051** | **0.054** | **0** | **0.06** | **0.269** | **0.006** | **0.003** | **0.053** | **0** | **0** | **0.01** | **0.100** | **0.114** |
|  | **GSM1629364** | **0** | **0.05** | **0.079** | **0.054** | **0** | **0.269** | **0** | **0.004** | **0** | **0.073** | **0** | **0.029** | **0.004** | **0** | **0.152** | **0.237** | **0.015** | **0** | **0.028** | **0** | **0** | **0.007** | **0.060** | **0.153** |
|  | **GSM1629365** | **0** | **0.05** | **0.113** | **0.058** | **0** | **0.243** | **0** | **0** | **0.05** | **0** | **0.001** | **0.019** | **0.016** | **0** | **0.12** | **0.249** | **0.022** | **0** | **0.052** | **0** | **0** | **0.009** | **0.410** | **0.032** |
|  | **GSM1629366** | **0.033** | **0.031** | **0.094** | **0.053** | **0** | **0.138** | **0** | **0.01** | **0.067** | **0.029** | **0.043** | **0** | **0.005** | **0** | **0.098** | **0.352** | **0.01** | **0** | **0.025** | **0** | **0** | **0.012** | **0.170** | **0.071** |
|  | **GSM1629367** | **0.048** | **0.024** | **0.111** | **0.036** | **0** | **0.202** | **0** | **0** | **0.03** | **0** | **0.093** | **0** | **0.026** | **0** | **0.05** | **0.305** | **0.015** | **0** | **0.05** | **0** | **0** | **0.01** | **0.320** | **0.039** |
|  | **GSM1629368** | **0.002** | **0.03** | **0.113** | **0.131** | **0** | **0.081** | **0.006** | **0.004** | **0.04** | **0.076** | **0** | **0** | **0.012** | **0** | **0.117** | **0.333** | **0.013** | **0** | **0.03** | **0** | **0** | **0.012** | **0.040** | **0.231** |
|  | **GSM1629369** | **0** | **0.054** | **0.063** | **0.143** | **0** | **0.09** | **0** | **0** | **0.04** | **0.101** | **0** | **0.022** | **0** | **0** | **0.125** | **0.276** | **0.013** | **0** | **0.053** | **0** | **0** | **0.02** | **0.050** | **0.182** |
|  | **GSM1629370** | **0.037** | **0.011** | **0.131** | **0.055** | **0** | **0.028** | **0** | **0.037** | **0.022** | **0.093** | **0** | **0.009** | **0.011** | **0** | **0.288** | **0.244** | **0.018** | **0** | **0.008** | **0** | **0** | **0.007** | **0.000** | **0.499** |
