# Supplementary material for: Identifying potential biomarkers in hepatitis B virus infection and its response to the antiviral therapy by integrated bioinformatic analysis
Source: J Cell Mol Med. 2021 May 26;25(14):6558–72. doi: 10.1111/jcmm.16655 (PMC8278120; doi:10.1111/jcmm.16655)
Supplement: Supplementary file 6 — Table S5 [file JCMM-25-6558-s004.docx]

**Table S5. The ncRNA associated with hepatitis B identified by MNDR platform**

| ncRNA Symbol | ncRNA Category | Species | Disease Name | Score |
| --- | --- | --- | --- | --- |
| H19 | lncRNA | Homo sapiens | Hepatitis B/Hepatitis B, Chronic | 0.999141 |
| TP53COR1 | lncRNA | Homo sapiens | Hepatitis B/Hepatitis B, Chronic | 0.973352 |
| GAS5 | lncRNA | Homo sapiens | Hepatitis B | 0.982118 |
| hsa-mir-101-1 | miRNA | Homo sapiens | Hepatitis B | 1 |
| hsa-mir-101-2 | miRNA | Homo sapiens | Hepatitis B | 1 |
| hsa-mir-122 | miRNA | Homo sapiens | Hepatitis B/Hepatitis B, Chronic | 1 |
| hsa-mir-15a | miRNA | Homo sapiens | Hepatitis B | 1 |
| hsa-mir-338 | miRNA | Homo sapiens | Hepatitis B | 1 |
| hsa-mir-132 | miRNA | Homo sapiens | Hepatitis B | 0.99998 |
| hsa-mir-141 | miRNA | Homo sapiens | Hepatitis B | 0.99998 |
| hsa-mir-16-1 | miRNA | Homo sapiens | Hepatitis B | 0.99998 |
| hsa-miR-499a-3p | miRNA | Homo sapiens | Hepatitis B | 0.99998 |
| hsa-miR-125b-5p | miRNA | Homo sapiens | Hepatitis B | 0.999424 |
| hsa-mir-501 | miRNA | Homo sapiens | Hepatitis B | 0.999424 |
| hsa-miR-106a-5p | miRNA | Homo sapiens | Hepatitis B | 0.999141 |
| hsa-miR-122-5p | miRNA | Homo sapiens | Hepatitis B | 0.999141 |
| hsa-miR-155-5p | miRNA | Homo sapiens | Hepatitis B | 0.999141 |
| hsa-mir-215 | miRNA | Homo sapiens | Hepatitis B | 0.999141 |
| hsa-mir-1-1 | miRNA | Homo sapiens | Hepatitis B | 0.99029 |
| hsa-mir-1-2 | miRNA | Homo sapiens | Hepatitis B | 0.99029 |
| hsa-mir-146a | miRNA | Homo sapiens | Hepatitis B | 0.99029 |
| hsa-mir-29a | miRNA | Homo sapiens | Hepatitis B | 0.982118 |
| hsa-mir-372 | miRNA | Homo sapiens | Hepatitis B | 0.982118 |
| hsa-mir-373 | miRNA | Homo sapiens | Hepatitis B | 0.982118 |
| hsa-mir-602 | miRNA | Homo sapiens | Hepatitis B | 0.973352 |
| hsa-mir-106b | miRNA | Homo sapiens | Hepatitis B | 0.888683 |
| hsa-mir-125a | miRNA | Homo sapiens | Hepatitis B | 0.795006 |
| hsa-mir-181a-1 | miRNA | Homo sapiens | Hepatitis B | 0.795006 |
| hsa-mir-181a-2 | miRNA | Homo sapiens | Hepatitis B | 0.795006 |
| hsa-mir-181b-1 | miRNA | Homo sapiens | Hepatitis B | 0.795006 |
| hsa-mir-181b-2 | miRNA | Homo sapiens | Hepatitis B | 0.795006 |
| hsa-mir-200a | miRNA | Homo sapiens | Hepatitis B | 0.795006 |
| hsa-mir-200b | miRNA | Homo sapiens | Hepatitis B | 0.795006 |
| hsa-mir-200c | miRNA | Homo sapiens | Hepatitis B | 0.795006 |
| hsa-mir-25 | miRNA | Homo sapiens | Hepatitis B | 0.795006 |
| hsa-mir-210 | miRNA | Homo sapiens | Hepatitis B/Hepatitis B, Chronic | 0.694506 |
| hsa-let-7c | miRNA | Homo sapiens | Hepatitis B | 0.694506 |
| hsa-miR-101-3p | miRNA | Homo sapiens | Hepatitis B | 0.694506 |
| hsa-mir-125b | miRNA | Homo sapiens | Hepatitis B | 0.694506 |
| hsa-mir-150 | miRNA | Homo sapiens | Hepatitis B | 0.694506 |
| hsa-miR-192-5p | miRNA | Homo sapiens | Hepatitis B | 0.694506 |
| hsa-mir-197 | miRNA | Homo sapiens | Hepatitis B | 0.694506 |
| hsa-miR-210-3p | miRNA | Homo sapiens | Hepatitis B | 0.694506 |
| hsa-mir-22 | miRNA | Homo sapiens | Hepatitis B | 0.694506 |
| hsa-mir-223 | miRNA | Homo sapiens | Hepatitis B | 0.694506 |
| hsa-mir-23b | miRNA | Homo sapiens | Hepatitis B | 0.694506 |
| hsa-miR-29a-3p | miRNA | Homo sapiens | Hepatitis B | 0.694506 |
| hsa-mir-583 | miRNA | Homo sapiens | Hepatitis B | 0.694506 |
| hsa-mir-663a | miRNA | Homo sapiens | Hepatitis B | 0.694506 |
| hsa-miR-99a-5p | miRNA | Homo sapiens | Hepatitis B | 0.694506 |

ncRNA：non-coding RNA；
