# Supplementary material for: Identifying potential biomarkers in hepatitis B virus infection and its response to the antiviral therapy by integrated bioinformatic analysis
Source: J Cell Mol Med. 2021 May 26;25(14):6558–72. doi: 10.1111/jcmm.16655 (PMC8278120; doi:10.1111/jcmm.16655)
Supplement: Supplementary file 7 — Table S6 [file JCMM-25-6558-s005.docx]

**Table S6. Clinical characteristics of included patients at baseline**

| Items | Nonresponder(n=12) | Responder(n=15) | *P* | *Z****/t*** |
| --- | --- | --- | --- | --- |
| Age(years,mean(SD)) | 8.82(2.76) | 6.10(4.50) | 0.08 | -2.24 |
| Weight(kg,median(IQR)) | 28.75(21.25-35.38) | 18.50(15.00-24.00) | 0.06 | -1.90 |
| Sex(male/female,n1/n2) | 10/2 | 10/5 | 0.41 | / |
| Therapeutic regimen(entecavir vs pegylated interferon, n1/n2) | 7/5 | 8/7 | 1.00 | / |
| Pretreatment viral load  ((Log10 HBV-DNA,IU/ml,median(IQR)) | 8.09(7.38-8.47) | 7.35(7.11-7.61) | 0.07 | -2.05 |
| Pretreatment ALT(U/L,mean(SD)) | 165.24(33.43) | 209.67(33.60) | 0.50 | -0.93 |
| Pathology: inflammatory grade (G0-1/G≥2, n1/n2) | 4/8 | 7/8 | 0.70 | / |
| Pathology: fibrosis stage (S0-1/S≥2, n1/n2) | 6/6 | 7/8 | 1.00 | / |
| CCL4(pg/ml,median(IQR)) | 65.12(47.73-89.04) | 108.02(89.10-150.19) | <0.05 | -2.05 |
| CCL5(pg/ml,median(IQR)) | 11026.48(10443.57-11711.52) | 11634.76(10679.85-11931.45) | 0.20 | -1.32 |
| CXCL9(pg/ml,median(IQR)) | 72.90(30.92-89.30) | 127.62(35.51-231.57) | 0.28 | -1.12 |
| CXCL10(pg/ml,median(IQR)) | 225.47(136.64-439.23) | 495.54(282.77-844.15) | <0.05 | -2.15 |
| CXCL13(pg/ml,median(IQR)) | 13.93(9.39-22.16) | 20.40(16.19-28.14) | 0.08 | -1.81 |
